# Mathematical modeling of unsteady flow with uniform/non-uniform temperature and magnetic intensity in a half-moon shaped domain

**DOI:** 10.1016/j.heliyon.2022.e09015

**Published:** 2022-03-01

**Authors:** Tarikul Islam, N. Parveen, R. Nasrin

**Affiliations:** a Department of Mathematics, Bangladesh University of Engineering and Technology, Dhaka-1000, Bangladesh; b Department of Mathematics, Bangabandhu Sheikh Mujibur Rahman Science and Technology University, Gopalganj-8100, Bangladesh

**Keywords:** Mathematical modeling, Unsteady flow, Uniform/non-uniform temperature, Magnetic intensity, Nanofluids, Half-moon shaped domain

## Abstract

The mathematical modeling of two-dimensional unsteady free convective flow and thermal transport inside a half-moon shaped domain charged in the presence of uniform/non-uniform temperature and magnetic effects with Brownian motion of the nanoparticles has been conducted. Thirty-two types of nanofluids in a combination of various nanoparticles and base fluids having different sizes, shapes, and solid concentrations of nanoparticles are chosen to examine the better performance of heat transfer. The circular boundary is cooled while the diameter boundary is heated with uniform/non-uniform temperature. An external uniform/non-uniform/periodic magnetic field is imposed along diameter. The powerful partial differential equations solver, finite element method of Galerkin type, has been engaged in numerical simulation. The numerical solution's heat transfer mechanism reaches a steady state from the unsteady situation within a very short dimensionless time of about 0.65. The thermal transport rate enhances for increasing buoyancy force whereas decreases with higher magnetic intensity. The uniform thermal condition along the diameter of half-moon gives a higher thermal transport rate compared to non-uniform heating conditions. The non-uniform magnetic field provides greater values of the mean Nusselt number than the uniform field. In addition, the outcomes also predict that a better rate of temperature transport for kerosene-based nanofluid than water-based, ethylene glycol-based, and engine oil-based nanofluid. The heat transfer rate is observed at about 67.86 and 23.78% using Co-Kerosene and Co-water nanofluids, respectively, with an additional 1% nanoparticles volume fraction. The blade shape nanoparticles provide a better heat transfer rate than spherical, cylindrical, brick, and platelet shapes. Small size nanoparticles confirm a higher value of average Nusselt number than big size. Mean Nusselt number increases 22.1 and 5.4% using 1% concentrated Cu-water and Cu-engine oil nanofluid, respectively than base fluid.

## Nomenclature

a: wave amplitude
$B_{\text{0}}$: Magnitude of magnetic field kg s^−2^A^−1^
cp: Specific heat at constant pressure J kg^−1^K^−1^
g: Gravitational acceleration m s^−2^
Ha: Hartmann number
H: Height of the cavity
k: Thermal conductivity W m^−1^K^−1^
L: Length of the enclosure at bottom wall
Nuav: Average Nusselt number
p: Dimensional pressure kg m^−1^s^−2^
P: Dimensionless pressure
K: Wave number
*Pr*: Prandtl number
Ra: Rayleigh number
T: Fluid temperature K
t: Dimensional time s
*u, v*: Dimensional velocity components m s^−1^
*U, V*: Dimensionless velocity components
*x, y*: Dimensional coordinates m
*X, Y*: Dimensionless coordinates

**Greek symbols**
α: Thermal diffusivity m^2^s^−1^
β: Thermal expansion coefficient K^−1^
*δ*: Dependent dimensionless variable
ϕ: Volume fraction of nanoparticles
μ: Dynamic viscosity kg m^−1^s^−1^
υ: Kinematic viscosity m^2^s^−1^
τ: Dimensionless time
θ: Non-dimensional temperature
ρ: Density kg m^−3^
σ: Electric conductivity
*ψ*: Stream function
λ: Period of the magnetic field

**Subscript**
h: Hot surface
c: Cold surface
*nf*: Nanofluid
*sp*: Solid particle
*bf*: Base fluid
L: Local

## 1 Introduction

Free convection within different cavities has been obtained significant consideration due to its direct engineering applications such as solar engineering applications, geophysical fluid mechanics, nuclear reactor cooling, electrical systems, fire engineering, enhancing cooling systems in the vehicle, heat exchangers, petroleum reservoirs, and so on. The main advantages of natural convection cooling systems are their simplicity, low noise, and minimum cost. Different researchers [1], [2], [3], [4], [5], [6], [7], [8] have performed various experimental and numerical studies to properly understand the free convection temperature transport phenomenon. Oztop and Nada [9] investigated the characteristics of the free convective flow of nanofluids inside a rectangle chamber numerically. The outcomes showed that the highest temperature transport was also observed for copper nanoparticles. Ghasemi et al. [10] investigated the effect of magnetic intensity on free convection in a square vessel using water-alumina nanofluid. Aminossadati and Ghasemi [11] also studied the enhancement of the natural convective flow of nanofluids within an isosceles triangle shape enclosure. They found that thermal performance enhances with the increase in the Rayleigh number within the chamber. Saleh et al. [12] performed free convective temperature transport within the trapezoidal cavity, containing copper-water and Al_2_O_3_-water nanofluids. They presented that the heat transfer rate enhances more with copper nanoparticles.

Buoyancy initiated natural convective fluid flow, and temperature transport is a significant phenomenon in science and engineering for its numerous potential applications such as electronic cooling, electronics, heat exchangers, automotive, etc. Buoyancy forces and temperature differences are the leading causes of natural convective heat transfer. Rashmi et al. [13] researched free convective temperature transport using Al_2_O_3_-water nanofluids. Sheikhzadeh et al. [14] investigated the heat transport and buoyancy-driven fluid flow within a rectangle chamber that contains TiO_2_–water nanofluid. Arani et al. [15] studied the numerically free convective flow of laminar and incompressible Ag-water nanofluids in the square chamber. Solemani et al. [16] performed the natural convective temperature transfer of Cu–water nanofluid in the semi-annulus cavity. Nasrin and Parvin [17] analyzed the temperature transport mechanism of free convection within a trapezoidal enclosure containing copper-water nanofluid. The results showed that nanoparticles volume fraction significantly impacts heat transfer. Sheikholeslami et al. [18] researched MHD (magnetohydrodynamic) free convection using Cu-water nanofluid within an inclined half annulus based on the finite element method. Nasrin and Alim [19] performed free convective heat transport of nanofluids inside the cavity by two different nanoparticles. Hussain and Hussain [20] investigated heat transfer enhancement on free convective within a parallel shape cavity using Cu-water nanofluid.

Nowadays, Nanofluids are commonly engaged in the enhancement of temperature transport because of their enriched thermo-physical properties. Different particles such as Al_2_O_3_, Cu, TiO_2_, Fe_3_O_4_, CuO, Co, Fe_2_O_3_, silver, silicon, carbon nanotubes are available commercially. Water, kerosene, engine oil, pump oil, kerosene, and so on are used widely as conventional fluids. Malvandi et al. [21] performed the convective temperature transport within an annulus using Al_2_O_2_-water nanofluid. Rahman and Al-Hatmi [22] investigated a comprehensive study about the characteristics of magneto-hydrodynamics temperature flow. The results showed that the heat transfer rate is higher in TiO_2_-water nanofluid compared to the heat transfer rate in Al_2_O_3_-water and Cu-water nanofluids. Koopaee and Jelodari [23] investigated the impact of the inclination angle of magnetic field on time-dependent free convective temperature transport of nanofluids within an enclosure where Al_2_O_3_ was used as nanoparticles. Mejri et al. [24] performed the magnetic effect on the laminar free convective flow of Al_2_O_3_ nanoparticles. The heat transfer rate decreases with the increase of nanoparticles. Sheikholeslami et al. [25] performed MHD effects on CuO–water nanofluid flow and heat transfer with Brownian motion. This study predicts that the Rayleigh number enhances heat transfer whereas decreases with a higher Hartmann number. Rahman et al. [26] investigated time-dependent MHD convection using Cobalt–kerosene ferrofluid within a semi-circular cavity employing finite element analysis.

Rahman et al. [27] performed free convective temperature flow of CNT-water nanofluid. The outcomes predict that the nanoparticle volume fraction can control the flow field and temperature distribution. Alsabery et al. [28] performed the free convective flow of nanofluids within the square chamber. The results show that sinusoidal temperature variations significantly enhance the convection heat transfer rate. Uddin et al. [29] performed a fundamental and comprehensive study of the nanofluids. This study narrated the fundamental concepts of nanofluids and properties and the potential application and advantages of nanofluids in various sectors widely. Nasrin et al. [30], [31] performed MHD free/conjugate heat transfer in circular/arc/rectangular cavities filled with different nanofluids. Weheibi et al. [32] analyzed the free convective temperature flow of nine different nanofluids, including various shapes of the nanoparticles within a trapezoidal enclosure. This research shows the solution becomes the steady-state with a strong buoyancy force, and the highest temperature transport rate was observed for cobalt-engine oil nanofluid and blade shape nanoparticles. Qi et al. [33] researched free convective heat transport of Cu/diamond–gallium nanofluid in the rectangle chamber. The results show that heat transport can be improved by 73.0% with Cu–Ga nanofluid than liquid metal gallium at the low-temperature difference (ΔT=1K).

The science which conducts the reciprocal interaction of the conducting liquid and magnetic field is known as MHD. Various investigations have been done on MHD regarding different geometry and various boundary conditions. MHD has many applications like crystal process, solar technologies, boiler, manufacturing technology, chemical and food processing, etc. MHD convection plays a vital role in materials engineering. Ouyahia et al. [34] performed the MHD heat performance of titanium dioxide nanoparticles within a triangle cavity. The magnetic effects upon the free convective of ferrofluid/nanofluid within the half-moon/closed-shaped enclosure were studied by [35], [36]. Mehryan et al. [37] performed the horizontal magnetic effects on the convective flow of ferrofluid within the square enclosure. They predicted that the magnetic field period enhances the intensity of convective flow and heat transfer rate.

The performance of temperature transport in many engineering procedures is an essential issue from an energy-saving perspective. The use of nano-sized particles can fulfill this desired target of thermal performance. For example, the thermal conductivity is 700 times greater for copper instead of water and 3000 times greater than engine oil. Kalbani and Rahman [38] also investigated the effects of MHD on the convective flow of nanofluid. Balushi et al. [39] researched the unsteady natural convectional flow of magnetic nanoparticles. Mahian et al. [40] performed a comprehensive study of nanofluid, its mathematical modeling and numerical simulation. Uddin and Rahman [41] also performed finite element analysis of free convection-flowing nanofluids. Uddin and Rasel [42] investigated the unsteady free convective flow of nanofluid. Izadi et al. [43] performed the periodic magnetic effects on the free convective flow of hybrid nanofluids within a permeable chamber. Marzougui et al. [44] performed MHD convective flow of copper-water nanofluids within an enclosure. They showed that heat transmission and flow field affect significantly by nanoparticle volume fraction and Hartmann number. Giwa et al. [45] researched the temperature performance of nanofluids by magnetic effects.

The lack of research observed in the literature of this study including:

• A very little research has been performed on the non-uniform magnetic fields, although it has many industrial and engineering applications.
• No research has been done to investigate the effect of vertical periodic magnetic field and its period in the semi-circular enclosure.
• Lack of enough experimental data of nanofluids, although it has superior thermophysical properties.
• Lack of sufficient data concerning the effect of nanoparticles Brownian motion, size, and shape.

This numerical analysis aims to examine the unsteady natural convection flow and heat transfer of nanofluids in a semi-circular enclosure under the influence of a vertical periodic magnetic field. Considering the importance of the thermal performance of nanofluids with non-uniform magnetic effects in different engineering processes such as crystallization, silver decomposition, heat exchanger, water evaporation, and so on, this investigation contributes some novelties as:

• Influence of a non-uniform magnetic field has been analyzed numerically.
• Impact of vertical periodic magnetic effect on natural convection has been done to confirm its applicability in thermal engineering processes.
• Investigating the effect of periodic force generated by the periodic magnetic field on heat transfer and fluid flow.
• Investigating a comparison effect of a wide range of nanoparticles and base fluid.
• Comparative study on the thermal performance of various nanofluids with different thermal boundary conditions.
• Investigating the contribution of nanoparticles Brownian motion on the thermal system.
• Investigating the effect of nanoparticles size and shape factor.

## 2 Physical modeling

A time-dependent, incompressible, laminar, two-dimensional free convective flow of nanofluids within the half-moon-shaped domain has been considered for mathematical modeling. The bottom diameter of the cavity represents the *x*-axis and *y*-axis normal to it. The cavity walls are considered as fixed and natural convection has been introduced by making temperature differences between heated and cold sidewalls.

The circular wall is cooled at low-heat $T_{c}$, whereas the bottom diameter is heated. Thus, the thermal boundary conditions (TBC) at the diameter are as:

Case I: uniform temperature $T_{h}$ ($T_{c}<T_{h}$)
Case II: linear temperature = $T_{C}+(T_{h}-T_{c})(1-\frac{x}{L})$,
Case III: non-uniform temperature $=T_{C}+(T_{h}-T_{c})(\frac{x}{L})(1-\frac{x}{L})$,
Case IV: sinusoidal/periodic temperature $=T_{C}+(T_{h}-T_{c})(\frac{a}{L})sin(Kx)$,
Case V: square of sinusoidal temperature $=T_{C}+(T_{h}-T_{c})(\frac{a}{L})sin^{2}(Kx)$,

where, *a* represents wave amplitude, and *K* represents wave number, where K=2π/L.

An external uniform/non-uniform/periodic magnetic field intensity has been engaged as a sinusoidal function of the *x*-coordinate. The relation of the vertical periodic magnetic intensity is denoted as:

(1) $$B=B_{0}sin(\frac{2\pi x}{\lambda_{0}})$$

where $\lambda_{0}$ represents the magnetic field period and represents the amplitude of the non-uniform periodic magnetic field.

**Figure 1:**
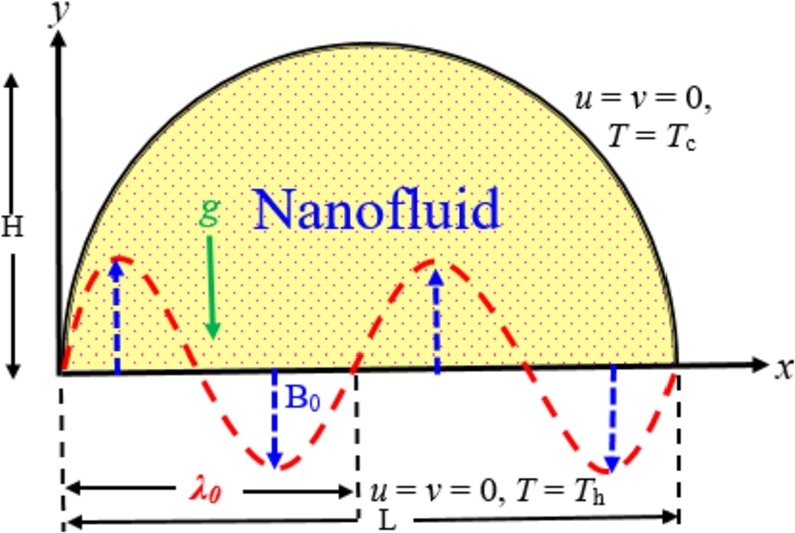
Schematic view of the half-moon shaped domain. (For interpretation of the colors in the figure(s), the reader is referred to the web version of this article.)

It is mentioned that the nano-sized particles are dispersed into the conventional fluid homogenously. The thermal equilibrium and thermal slip exist between the base fluids and nanoparticles. It is also mentioned that the physical property density in the buoyancy term varies among the thermo-physical properties of nanofluid while other properties remain constant during convection. Since the temperature difference is limited between cold and hot walls, this condition is reasonable. The gravitational acceleration works in the negative direction along the *y*-axis. All the solid boundaries are assumed to be rigid no-slip walls. 32-types of different nanofluids are also considered in this investigation. The geometry and coordinate systems are schematically shown in Fig. 1. The thermo-physical properties of various nanoparticles and base fluids are listed in Table 1.

**Table 1:** Thermo-physical properties of the various base fluids and solid particles.

Base Fluid/ Nanoparticles	$c_{p}$ [Jk g^−1^K^−1^]	*P* [kg m^−3^]	*K* [W m^−1^K^−1^]	*μ* [kg m^−1^s^−1]^	$\beta \times 10^{-5}$ [$K^{-1}$]	*σ* [Sm^−1^]	*Pr*
Water (H_2_O)	4179	997.1	0.613	0.001003	21	5.50 × 10^−6^	6.84
Kerosene	2090	780	0.149	0.00164	99	6.0 × 10^−10^	23
Ethylene Glycol	2382.1	1117.48	0.2492	0.022	57	1.07 × 10^−8^	210.3
Engine oil (EO)	1880.3	888.23	0.145	0.8451	70	23.004	10958.9
Copper (Cu)	385	8933	400	-	1.67	5.96 × 10^7^	-
Alumina (Al_2_O_3_)	765	3970	40	-	0.85	3.50 × 10^7^	-
Co	420	8900	100	-	1.3	1.602 × 10^7^	-
Fe_3_O_4_	670	5180	80.4	-	20.6	1.12 × 10^5^	-
TiO_2_	686.2	4250	8.9538	-	0.90	2.60 × 10^6^	-
Ag	233	10500	429	-	1.8	6.30 × 10^7^	-
Zn	387	7135	116	-	3.02	1.69 × 10^7^	-
CuO	531.8	6320	76.5	-	1.8	51.28 × 10^7^	-

## 3 Mathematical modeling

To drive the governing equations in dimensional form for the present study, applying the afore-mentioned considerations as follows:

Continuity equation:

(2) $$\frac{\partial u}{\partial x}+\frac{\partial v}{\partial y}=0$$

Momentum equation in *x*-direction:

(3) $$\frac{\partial u}{\partial t}+u\frac{\partial u}{\partial x}+v\frac{\partial u}{\partial y}=-\frac{1}{\rho_{nf}}\frac{\partial p}{\partial x}+\frac{\mu_{nf}}{\rho_{nf}}(\frac{\partial^{2}u}{\partial x^{2}}+\frac{\partial^{2}u}{\partial y^{2}})-\frac{1}{\rho_{nf}}\sigma_{nf}B_{0}^{2}\sin^{2}(\frac{2\pi x}{\lambda_{0}})u$$

Momentum equation in *y*-direction:

(4) $$\frac{\partial v}{\partial t}+u\frac{\partial v}{\partial x}+v\frac{\partial v}{\partial y}=-\frac{1}{\rho_{nf}}\frac{\partial p}{\partial y}+\frac{\mu_{nf}}{\rho_{nf}}(\frac{\partial^{2}v}{\partial x^{2}}+\frac{\partial^{2}v}{\partial y^{2}})+\frac{{\left(\rho \beta \right)}_{nf}}{\rho_{nf}}g(T-T_{c})$$

Energy equation:

(5) $$\frac{\partial T}{\partial t}+u\frac{\partial T}{\partial x}+v\frac{\partial T}{\partial y}=\alpha_{nf}(\frac{\partial^{2}T}{\partial x^{2}}+\frac{\partial^{2}T}{\partial y^{2}})$$

### 3.1 Initial and boundary conditions

The initial and boundary conditions of the above-narrated model are as follows:

(6) For t≤0; entire domain:u=0,v=0,T=0,p=0

(7) $$\text{For }t>0\text{; At the circular boundary:}\;u=0,v=0,T=T_{c}$$

At the diameter boundary:

(8) $$\text{Case I:}\;u=0,v=0,T=T_{h}$$

(9) $$\text{Case II:}\;u=0,v=0,T=T_{c}+(T_{h}-T_{c})(1-\frac{x}{L})$$

(10) $$\text{Case III:}\;u=0,v=0,T=T_{c}+(T_{h}-T_{c})(\frac{x}{L})(1-\frac{x}{L})$$

(11) $$\text{Case IV:}\;u=0,v=0,T=T_{c}+(T_{h}-T_{c})(\frac{a}{L})\sin(Kx)$$

(12) $$\text{Case V:}\;u=0,v=0,T=T_{c}+(T_{h}-T_{c})(\frac{a}{L})\sin^{2}(Kx)$$

### 3.2 Thermal and physical properties of nanofluids

To enhance the heat performance of nanofluids, the physical and thermal properties of nanofluids are essential. Nanofluids' material and thermal characteristics are listed as viscosity, density, thermal diffusivity, heat capacitance, thermal conductivity, and thermal expansion coefficient. For computing the physical and thermal properties of the nanofluids, the following formulas are used (Al-Weheibi et al. [32], Kalbani et al. [38], Uddin and Rahman [41]):

The effective viscosity of the nanofluids is expressed as follows:

(13) $$\mu_{nf}=\mu_{bf}\frac{1}{{\left(1-\phi \right)}^{2.5}}$$

The effective density of the nanofluid is expressed as follows

(14) $$\rho_{nf}=(1-\phi )\rho_{bf}+\phi \rho_{sp}$$

The thermal diffusivity of the nanofluid is expressed as follows

(15) $$\alpha_{nf}=k_{nf}/{\left(\rho c_{p}\right)}_{nf}$$

The heat capacitance of the nanofluid is given by

(16) $${\left(\rho c_{p}\right)}_{nf}=(1-\phi ){\left(\rho c_{p}\right)}_{bf}+\phi {\left(\rho c_{p}\right)}_{sp}$$

The Maxwell model of thermal conductivity is extended by including a shape factor by Hamilton and crosser [46] as follows:

(17) $$k_{nf}=\frac{k_{sp}+(n-1)k_{bf}-(n-1)(k_{bf}-k_{sp})\phi }{k_{sp}+(n-1)k_{bf}+(k_{bf}-k_{sp})\phi }k_{bf}$$

where *n* represents the nanoparticles shape factor.

The Brownian motion of nanoparticles has not been considered in the equation (17). But experimentally, it has been proved that the Brownian movement of nanoparticles plays an essential role in the heat transfer enhancement of nanofluids. Therefore, an appropriate model is considered for calculating thermal conductivity, including convectional static and Brownian parts. Therefore, nanofluids' thermal conductivity depends on nanoparticles volume fraction, the thermal conductivity of nanoparticles, temperature of the mixture, nanoparticles size, and base fluid properties considering viscosity and specific heat capacity.

(18) $$k_{nf}=k_{static}+k_{Brownian}$$

where $k_{\mathrm{static}}$ represents the static thermal conductivity based on Maxwell classical correlation and $k_{\mathrm{Brownian}}$ represents the dynamical part of nanofluids for the effect of Brownian motion on nanoparticles which is calculated as:

(19) $$k_{Brownian}=\frac{\phi \rho_{p}c_{p,p}}{2}\sqrt{\frac{2K_{B}T_{ref}}{3\pi d_{p}\mu_{nf}}}$$

where $K_{B}$ represents the Boltzmann constant and $d_{p}$ represents the diameter of nanoparticles.

The thermal expansion coefficient is expressed as follows

(20) $${\left(\rho \beta \right)}_{nf}=(1-\phi ){\left(\rho \beta \right)}_{bf}+\phi {\left(\rho \beta \right)}_{sp}$$

### 3.3 Dimensional analysis

The following dimensionless variables are introduced for the present study to convert the governing equations (2)–(5), including initial and boundary conditions (6)–(12) into the non-dimensional form:

(21) $$X=\frac{x}{L},\;Y=\frac{y}{L},\;A=\frac{a}{L},\;U=\frac{uL}{\alpha_{bf}},\;V=\frac{vL}{\alpha_{bf}},\;\theta =\frac{T-T_{c}}{T_{h}-T_{c}},\;P=\frac{pL^{2}}{\rho_{bf}\alpha_{bf}^{2}},\;\tau =\frac{t\alpha_{bf}}{L^{2}},\;\lambda =\frac{\lambda_{0}}{L}$$

Employing the equation (21) into (2)–(5) including initial and boundary conditions (6)–(12) as follows:

(22) $$\frac{\partial U}{\partial X}+\frac{\partial V}{\partial Y}=0$$

(23) $$\frac{\partial U}{\partial \tau }+U\frac{\partial U}{\partial X}+V\frac{\partial U}{\partial Y}=-\frac{\rho_{bf}}{\rho_{nf}}\frac{\partial P}{\partial X}+Pr(\frac{\mu_{nf}}{\mu_{bf}})(\frac{\rho_{bf}}{\rho_{nf}})(\frac{\partial^{2}U}{\partial X^{2}}+\frac{\partial^{2}U}{\partial Y^{2}})-a^{2}Pr(\frac{\rho_{bf}}{\rho_{nf}})(\frac{\sigma_{nf}}{\sigma_{bf}})\mathrm{si}n^{2}(\frac{2\pi X}{\lambda })U$$

(24) $$\frac{\partial V}{\partial \tau }+U\frac{\partial V}{\partial X}+V\frac{\partial V}{\partial Y}=-\frac{\rho_{bf}}{\rho_{nf}}\frac{\partial P}{\partial Y}+Pr(\frac{\mu_{nf}}{\mu_{bf}})(\frac{\rho_{bf}}{\rho_{nf}})(\frac{\partial^{2}V}{\partial X^{2}}+\frac{\partial^{2}V}{\partial Y^{2}})+\frac{{\left(\rho \beta \right)}_{nf}}{\rho_{nf}\beta_{bf}}RaPr\theta$$

(25) $$\frac{\partial \theta }{\partial \tau }+U\frac{\partial \theta }{\partial X}+V\frac{\partial \theta }{\partial Y}=(\frac{\alpha_{nf}}{\alpha_{bf}})(\frac{\partial^{2}\theta }{\partial X^{2}}+\frac{\partial^{2}\theta }{\partial Y^{2}})$$

The non-dimensional boundary condition becomes

(26) For τ=0, whole domain:U=0,V=0,θ=0,P=0

For τ>0, the dimensionless boundary conditions:

(27) At the circular wall:U=0,V=0,θ=0

At the bottom wall:

(28) Case I:u=0,v=0,θ=1

(29) Case II:u=0,v=0,θ=1−X

(30) Case III:u=0,v=0,θ=X(1−X)

(31) Case IV:u=0,v=0,θ=Asin⁡(2πX)

(32) $$\text{Case V:}\;u=0,v=0,\theta =A\sin^{2}(2\pi X)$$

where, A=a/L, $Ra=\frac{g\beta_{bf}(T_{h}-T_{c})L^{3}}{\upsilon_{bf}\alpha_{bf}}$, $Ha=B_{o}L\sqrt{\sigma_{bf}/\mu_{bf}}$, and $\mathrm{Pr}=\upsilon_{nf}/\alpha_{bf}$ represent the non-dimensional amplitude, Rayleigh number, Hartmann number and Prandtl number, respectively.

### 3.4 Calculation of Nusselt number

For this model, the important physical parameter quantities are local Nusselt number ($Nu_{L}$) and average Nusselt number ($Nu_{av}$) along the bottom heated wall of the cavity. The local Nusselt number is defined according to [47] as:

(33) $$Nu_{L}=\frac{Lq_{w}}{k_{bf}(T_{h}-T_{c})}$$

where the heat transfer from the bottom heated wall $q_{w}$ is given by

(34) $$q_{w}=-k_{nf}{\left(\frac{\partial \theta }{\partial Y}\right)}_{Y=0}$$

The average Nusselt number on the bottom heated wall of the cavity is expressed as

(35) $$Nu_{av}=-(\frac{k_{nf}}{k_{bf}})\int_{0}^{1}\frac{\partial \theta }{\partial Y}dX$$

## 4 Computational procedure

The semi-circular cavity has been discretized into numerous triangle elements in which the dimensionless governing equations (22)-(25), including boundary conditions (26)-(32), are employed for the numerical calculations. The finite element technique of the Galerkin weighted residual form has been employed for solving these problems. This numerical method has been narrated well in the book by Zienkiewicz and Taylor [48]. In this numerical method, the triangular elements of non-uniform type are constructed for the present geometric domain. The domain of the solution space is discretized into finite element meshes that are compressed of triangular elements of non-uniform style. In the current investigation, triangle shape components of six nodes are employed for improving finite element equations where all six nodes are connected with velocity and temperature. The nodes at the corner are merely associated with pressure. The matching of the pressure gradient has happened between momentum equations for continuity requirement and a shape function of lower-order selected for the pressure that is satisfied through the equation of continuity. The identical pressure is considered with linear elements, whereas it is non-continuous among the elements. After that, the technique of Galerkin weighted residual is appointed in the governing non-linear partial differential equations, which transfer the non-linear partial differential governing equations into a system of integral equations. Integral parts of these equations are accomplished employing Gauss's quadrature technique. After that, boundary conditions are also used to modify the non-linear algebraic equations. For solving these non-linear algebraic equations in matrix form, Newton-Raphson iteration is devoted. The convergent criteria of the numerical solution procedure have been estimated as $|\Gamma^{m+1}-\Gamma^{m}|\leq 10^{-5}$, where Γ represents subordinate variables (U,V,θ) and *m* is the number of iterations.

### 4.1 Grid independency test

**Figure 2:**
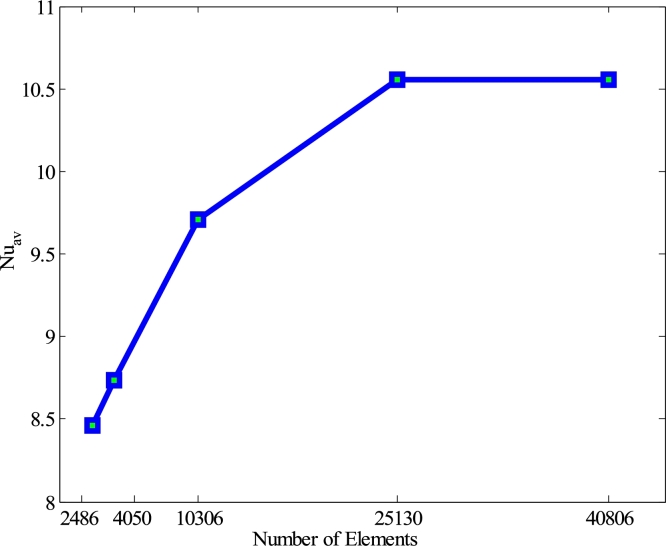
Convergence of average Nusselt number for various elements number for Cu-H_2_O nanofluid with TBC of case I.

For the grid-independent test, a comprehensive non-uniform grid sensitivity study is performed for the current problem when $Ra=10^{5}$, Ha=20, λ=0.5, ϕ=0.04, Pr=6.84, n=3, d=10 nm, and τ=1. Five different non-uniform grid systems containing elements numbers such as 2486, 4050, 10306, 25130, and 40806 are examined for the present semi-circular enclosure. For the number as mentioned earlier of elements, the design of the numerical calculation of mean Nusselt number ($Nu_{av}$) has been examined for checking the development of grid fineness which is shown in Fig. 2. The value of the mean Nusselt number for elements size 25130 depicts an ordinary difference with elements size 40806. Therefore, to get accurate results, the size of the elements 25130 and 40806 can be used. In this study, the size of the elements 25130 is employed for getting the grid-independent solution and computational time limits.

### 4.2 Code validation through streamlines and isotherms

**Figure 3:**
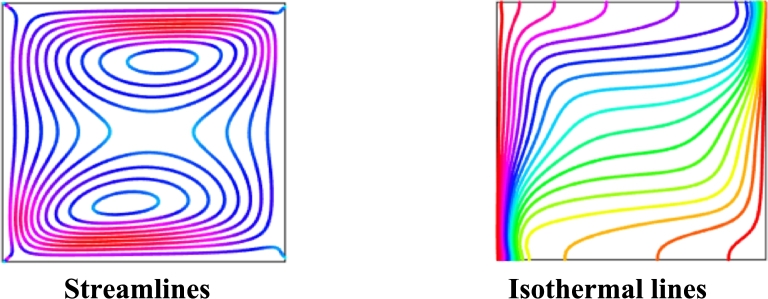
Code validation of current results in terms of streamlines and isothermal lines.

To access the correctness of our current numerical scheme, the results generated by the present numerical scheme have been compared with the outcomes of Mehryan et al. [37] using streamlines and isothermal lines considering uniform magenetic field with $Ra=10^{6}$, Ha=25, ϕ=0.04, and λ=1. Fig. 3 represents the results generated from current numerical code concerning streamlines and isothermal lines. These two figures are similar to 5(e) and 6(e), respectively in the research of Mehryan et al. [37] with above mentioned parametric values. The results show strong permission and boost the confidence for employing the current code.

## 5 Results and discussion

The simulated numerical results are analyzed to investigate the effects of Rayleigh number ($10^{3}\leq Ra\leq 10^{6}$), Hartman number (0≤Ha≤80), the solid volume fraction of nanoparticles (0≤ϕ≤0.1), period of the uniform/non-uniform/periodic magnetic intensity (0≤λ≤1), and the different size of nanoparticles (1nm≤d≤10 nm). The numerical calculations are expressed in terms of streamline contours, isothermal lines, and average Nusselt number. In the numerical simulations, four types of base fluid such as water (H_2_O), Engine Oil (EO), kerosene, and Ethylene Glycol (EG) and eight various types of nanoparticles such as Cu, Co, Fe_3_O_4_, Al_2_O_3_, TiO_2_, Ag, Zn, and CuO also considered checking the augmentation of temperature transport. Firstly, the numerical outcomes focus on streamlines and isotherms with non-dimensional time (*τ*) calculated for those as mentioned earlier different physical model parameters. Secondly, it is focused on heat transfer rate through the average Nusselt number along the heated bottom wall to investigate heat transport performance for various physical parameters. Furthermore, different types of nanofluids and uniform/non-uniform thermal boundary conditions (TBC of the case I, case II, case III, case IV, and case V) are also examined regarding the average Nusselt number on the heated bottom wall for copper-water nanofluid to calculate the augmentation of heat transfer performance. The range of non-dimensional temperature of the fluid in the isothermal contours is obtained from 0 to 1 for the thermal boundary condition of case I, II, V, from 0 to 0.5 for case III, and from −1 to 1 for case IV.

### 5.1 Time evolution of the solution

**Figure 4:**
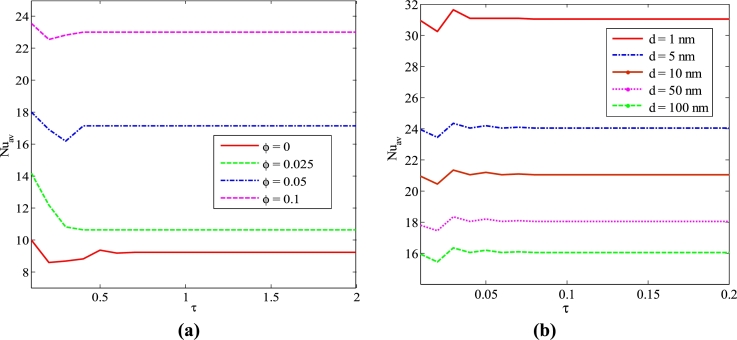
*Nu*_*av*_ against (a) *ϕ* and (b) *d* for different *τ* using Cu-H_2_O nanofluid for the case I.

Fig. 4 (a-b) represents the effects of different nanoparticles volume fractions and size of nanoparticles ($d_{p}$) on average Nusselt number on the heated bottom wall with dimensionless time for Cu-H_2_O nanofluid for uniform thermal boundary condition (TBC-case I) when Pr=6.84, Ha=20, $Ra=10^{5}$ and n=3. These figures show the average Nusselt number decreases initially and then reaches a steady state after a certain amount of time. The steady-state time is calculated approximately at τ=0.65 from these figures concerning different values of nanoparticle volume fraction. Fig. 4(a) indicates that the addition of nanoparticles into the base fluid significantly enhances the heat transfer rate. These figures depict that the solution takes more time to reach an unsteady state to state for the absence of nanoparticles in the base fluid. At the unstable flow, when the process is beginning, the average Nusselt number ($Nu_{av}$) is relatively higher, and it becomes constant after passing dimensionless time. In addition, the higher nanoparticles into the base fluid create a particle tapping; consequently, the characteristics of nanofluid may change from Newtonian to non-Newtonian. Therefore, the addition of nanoparticles into the base fluid assists the unsteady solution to reach a steady state. In the present numerical calculations, the volume fraction of nanoparticles has been varied from 0 to 10%. To check the qualitative change in the solution, we have used an extreme case of 10%. Fig. 4(b) shows that the average Nusselt number oscillates significantly for a certain initial period for different diameters of nanoparticles. After a certain time, the distributions of the average Nusselt number is the almost straight line which means that the solution reaches a steady-state for the diameter of nanoparticles. It is also observed that the mean Nusselt number oscillates more for the small size of particles compared to the large size of particles. Therefore, the small size of particles helps the solution to attain in steady than large nanoparticles because the settling velocity is negligible for the small size of nanoparticles. For getting a steady-state solution quickly, it can be assisted by the possible smaller size of nanoparticles.

**Figure 5:**
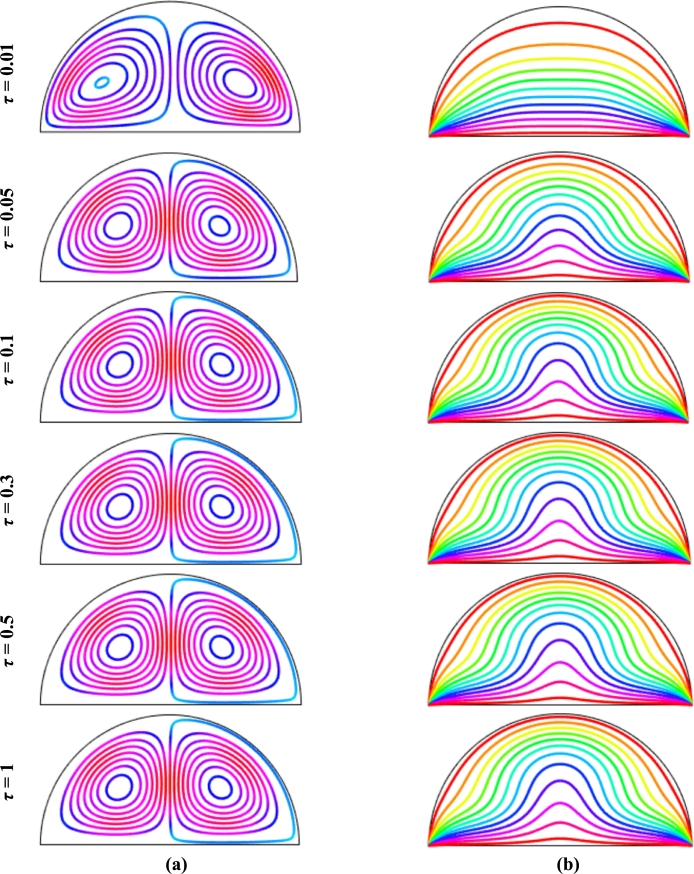
(a) Streamlines and (b) isotherms evaluation at different dimensionless time (*τ*) using Cu-H_2_O nanofluid for uniform thermal boundary condition (case I) with *Pr* = 6.84, *Ra* = 10^5^, *Ha* = 20, *d* = 10 nm, *n* = 3, *λ* = 0.5, and *ϕ* = 0.04.

Figs. 5(a-b) displays the evaluation of streamline contours and isotherms with non-dimensional time (*τ*) for uniform thermal boundary condition (case I) for Cu-H_2_O nanofluids when $Ra=10^{5}$, Ha=20, ϕ=0.04, and d=10 nm considering the time step Δτ=0.01. In a shorter time, it is seen that there are two symmetrical circulating vortices within the enclosure are formed where the eyes of the rotating cells of the streamlines near the heated wall. The rotating zone changes and intensifies at the heated wall and cooled wall. The eyes of the symmetrical circulation move to central circulation with the increase of non-dimensional time (*τ*), which indicates a higher velocity of the flow. For the increases of dimensionless time (*τ*), the streamlines pattern shows no significant changes until it reaches to steady state.

Fig. 5(b) shows, at τ=0.01, the flow is unsteady, and the isothermal lines are concentrated near the hot bottom wall that represents a higher temperature gradient due to buoyancy effects. As dimensionless time (*τ*) increases, the isothermal lines move upward at the middle of the diameter, representing higher heat flow in that region. The strength of the isothermal line grows with the non-dimensional time until it reaches a steady state. In addition, the isothermal lines change over time and display a marginal variation until it reaches the steady state.

### 5.2 Effect of uniform/non-uniform magnetic intensity

**Figure 6:**
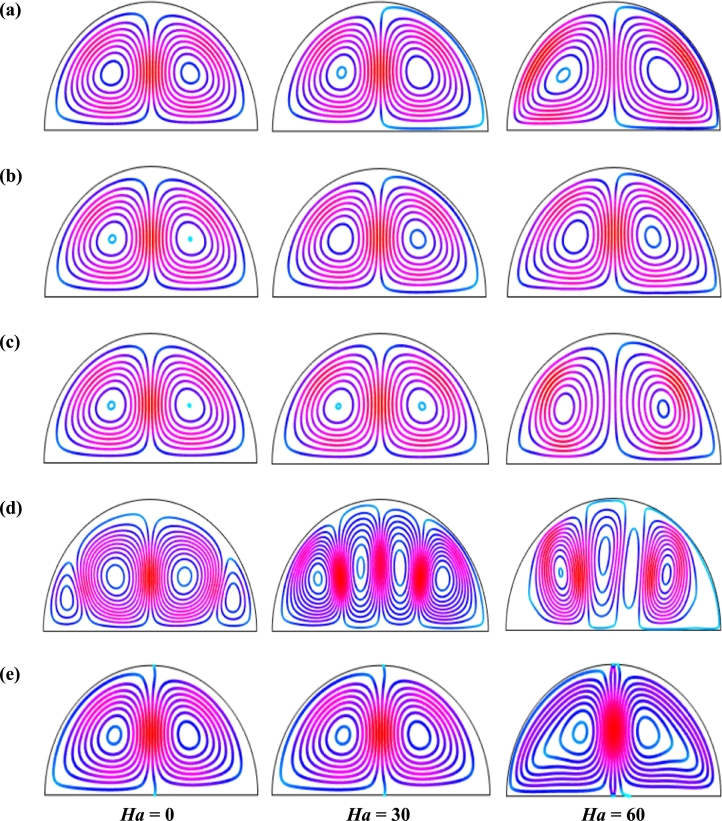
Effect of magnetic intensity on streamlines for (a) uniform magnetic field (umf), (b) *λ* = 0.1, (c) *λ* = 0.25, (d) *λ* = 0.5 and (e) *λ* = 1 using Cu-H_2_O nanofluid for uniform thermal boundary condition (case I) with *Ra* = 10^5^, *ϕ* = 0.04, *d* = 10 nm, *n* = 3, *Pr* = 6.84, and *τ* = 0.1 (unsteady case).

Figs. 6, respectively, represent the influence of streamlines for different Hartmann number (*Ha*) on the unsteady state (τ=0.1) for Cu-H_2_O nanofluid for uniform thermal system (case I) when Pr=6.84, $Ra=10^{5}$, d=10 nm, and n=3. These figures represent the evaluation of streamlines under the strong magnetic field. Two symmetrical vortices are observed for both uniform and non-uniform magnetic effects except the magnetic field period, λ=0.5. The strength of the flow diminishes with a stronger applied magnetic field, i.e., increase of Hartmann number. A strong field is imposed over the moving fluid by imposing an external applied magnetic field that has magnetic impressionability. The Lorentz force generated by magnetic field has a nature to oppose the varying its generation in fluid movement. This force field weakens the streams inside the cavity. Fig. 6 represents the pattern of the streamline contours when the system reaches its steady state. A little change in the strength of the streamline contours is observed with time. In addition, at λ=0.5, the intensity of the pattern of the streamline contours increases within the cavity.

**Figure 7:**
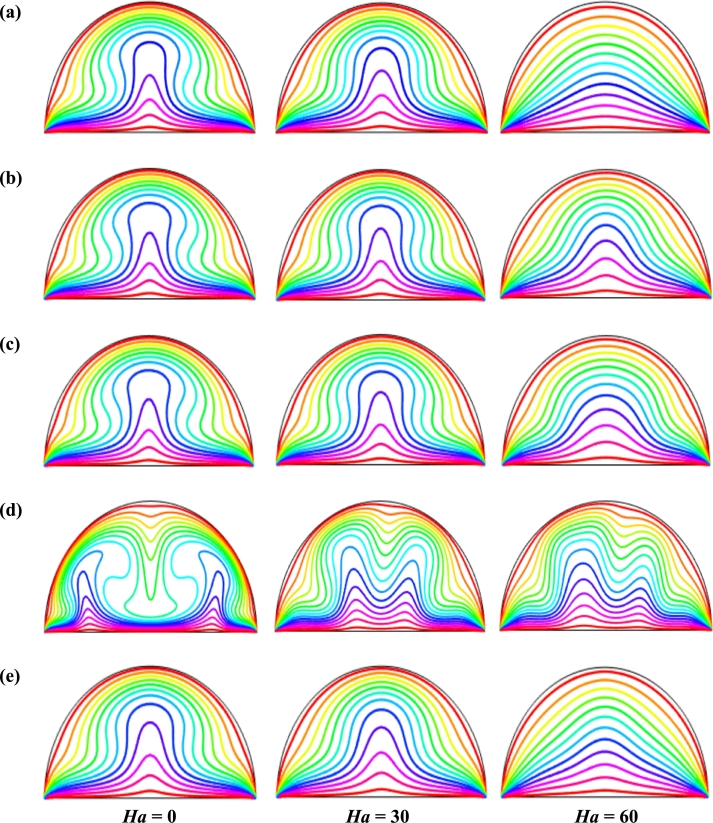
Effect of magnetic intensity on isothermal lines for (a) uniform magnetic field (umf), (b) *λ* = 0.1, (c) *λ* = 0.25, (d) *λ* = 0.5 and (e) *λ* = 1, for Cu-H_2_O nanofluid for uniform thermal boundary condition (case I) when *Ra* = 10^5^, *ϕ* = 0.04, *d* = 10 nm, *Pr* = 6.84, *n* = 3 and *τ* = 0.1 (unsteady case).

Figs. 7 exhibits the effect of isothermal lines for various Hartmann number (*Ha*) and period of the non-uniform magnetic field when Pr=6.84, $Ra=10^{5}$, d=10 nm, n=3, and τ=0.1 for Cu-H_2_O nanofluid for uniform thermal boundary condition (case I). These figures show that the pattern of the isothermal lines is almost like the uniform magnetic field and low period of the non-uniform magnetic effect (τ=0.1). These figures also show that isothermal lines are distorted neighbor the warmed diameter for the absence of the Hartmann number. But, for a higher magnetic effect (Ha=60), the isotherms converted very nearly parallel to the hot bottom wall, which indicates the dominance of conduction near the hot wall. This pattern of streamlines also indicates Hartmann number (*Ha*) doesn't influence the flow field greatly but also retards the thermal field within the cavity. In addition, the upper Hartmann number (*Ha*) is acting against convection within the enclosure. Moreover, the period of the magnetic field has a significant impact on fluid flow. The pattern of the isothermal lines changes with the changes of the period of the magnetic field.

**Figure 8:**
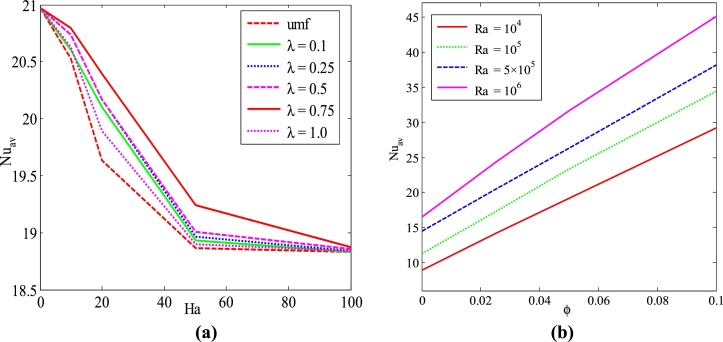
*Nu*_*av*_ against (a) uniform magnetic field (umf) and (b) *ϕ* for different periods of the magnetic field (*λ*) and Rayleigh number using Cu-H_2_O nanofluid with TBC of the case I.

Fig. 8(a-b) shows the mean Nusselt number against different Hartmann number (*Ha*) and volume fraction (*ϕ*) for the effect of uniform and non-uniform magnetic effect with the various period (*λ*) and Rayleigh number (*Ra*) for Cu-H_2_O nanofluid when Pr=6.84, $Ra=10^{5}$, and d=10 nm with uniform thermal boundary system (case I). Fig. 8(a) shows heat transport rate diminishes with the increase of Hartmann (*Ha*). This is because the Lorentz forces are increased by a higher Hartmann number (Ha=60), which produces a stronger resistance against the fluid movement. This reduces the thermal efficiency of the nanofluid flow and temperature transport rate. In addition, the period of the magnetic field plays a significant role in heat transport. It is noticed that a higher average rate of temperature transport is observed for the non-uniform magnetic effect when λ=0.75. Moreover, it is interesting to observe that a higher rate of heat transfer is noticed for non-uniform magnetic than uniform magnetic effect. It also observed that non-uniform magnetic fields conform better heat transfer rate. For the sinusoidal function of the magnetic field, an encounter periodic force field is created which reduces the heat transfer rate. The magnetic field can create an encounter physical environment that can affect any chemical and physical processes. The magnetic field is useful in material processes, heat exchangers, and various scientific research. The periodic magnetic field can create a periodic force. This periodic magnetic field is used in water evaporation, silver deposition, and protein crystallization, etc. The encountered periodic forces of the sinusoidal magnetic field are the key to the differences in temperature between the sinusoidal magnetic field and uniform magnetic field.

Fig. 8(b) illustrates that the average Nusselt number is significantly higher for a higher value of nanoparticles volume fraction and upper value of buoyancy force. The heat transfer rate increases monotonically with the increase of nanoparticle volume fraction. The heat transfer rate is more pronounced and increases rapidly for a higher *Ra*. In addition, heat transfer augmentation is more significant for nanofluids than base fluid.

### 5.3 Effect of buoyancy force

**Figure 9:**
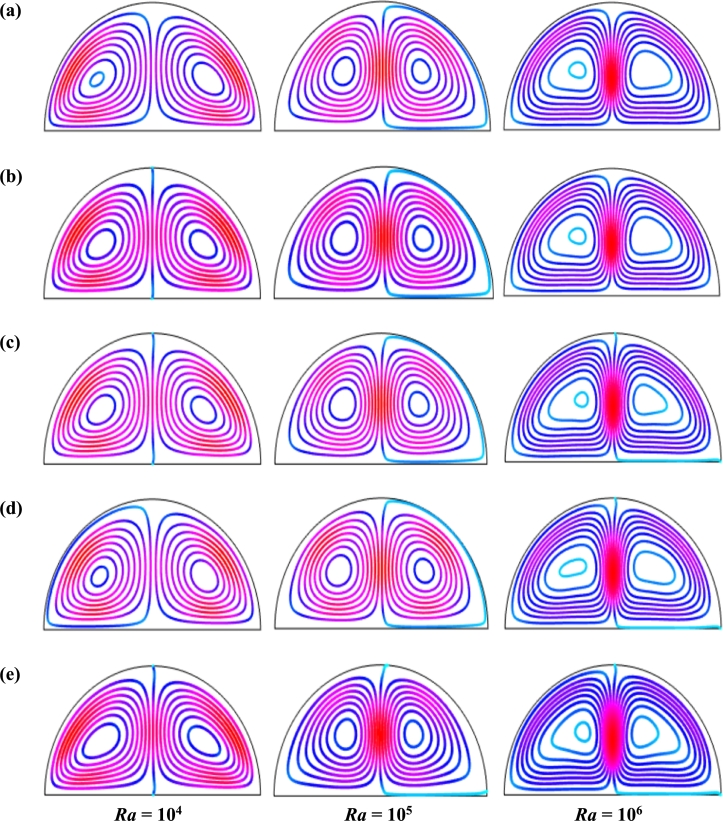
Effect of buoyancy force on streamlines for (a) uniform magnetic field (umf), (b) *λ* = 0.1, (c) *λ* = 0.25, (d) *λ* = 0.5, and (e) *λ* = 1 using Cu-H_2_O nanofluid for uniform TBC (case I) with *Ha* = 20, *ϕ* = 0.04, *d* = 10 nm, *n* = 3 and *τ* = 1 (steady case).

Fig. 9 represents the effect of buoyancy force Rayleigh number ($10^{4}\leq Ra\leq 10^{6}$) on streamlines respectively for steady case (τ=1) for Cu-H_2_O nanofluid for case I when ϕ=0.04, d=10 nm, Ha=20, Pr=6.84, and n=3. This result indicates that for all Rayleigh number (*Ra*), the buoyancy-driven rotating flows in the cavity is obvious. For small *Ra* ($=10^{4}$), two symmetrical circulation cell is seen inside the cavity for dominant characteristics of the flow field. As the Rayleigh number rises, the streamlines change and streamline contours intensify, which indicates a higher velocity gradient and strength in the natural convection. The isothermal lines condense near the heated bottom wall and circular cooled wall representing a higher temperature gradient. At a higher Rayleigh number ($Ra=10^{6}$), the streamlines circulation is more pronounced. At $Ra=10^{6}$, and λ=0.5, the more vortices are observed inside the enclosure. Furthermore, the streamlines pattern changes with the changes of the period of the magnetic field.

**Figure 10:**
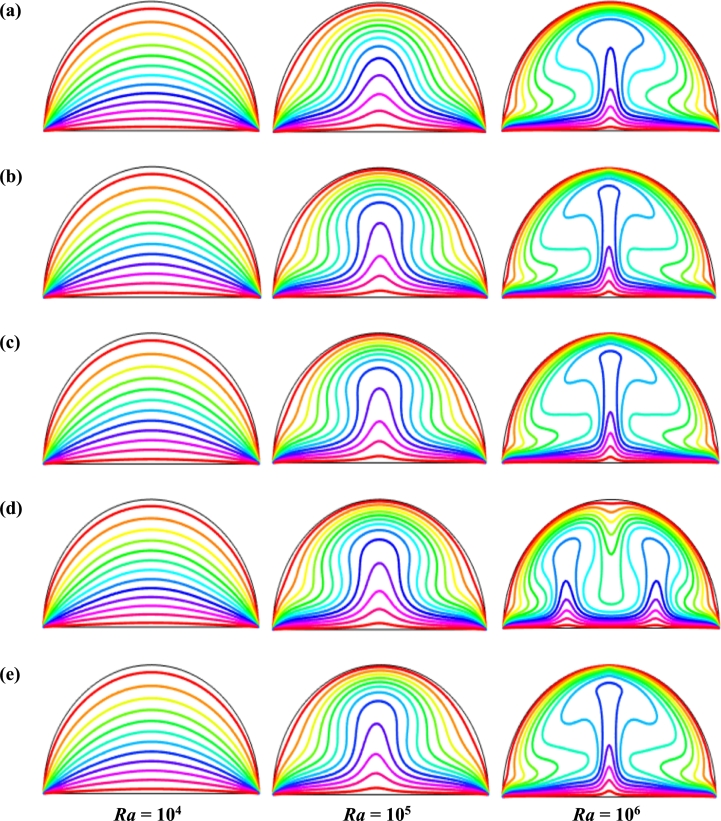
Effect of buoyancy force on isotherms for (a) uniform magnetic field (umf), (b) *λ* = 0.1, (c) *λ* = 0.25, (d) *λ* = 0.5, and (e) *λ* = 1 using Cu-H_2_O nanofluid for uniform TBC (case I) when *Ha* = 20, *ϕ* = 0.04, *d* = 10 nm, *n* = 3 and *τ* = 1 (steady case).

Figs. 10 represents the effects of Rayleigh number ($10^{4}\leq Ra\leq 10^{6}$) on isothermal lines respectively for steady case (τ=1) for uniform thermal systems (case I) when d=10 nm, Ha=20, Pr=6.8377, n=3, and ϕ=0.04 for Cu-H_2_O nanofluid. The strength of the fluid currents enhances with Rayleigh number (*Ra*) due to the influence of buoyant forces which increase the convective force. At the low Rayleigh number ($Ra=10^{4}$), the isothermal lines are almost parallel to each other to the heat source wall due to the weaker convection inside the cavity. Therefore, conduction is the significant mood of temperature transport for the lower buoyancy-driven parameter. The streamlines form a cavity-like arc near the top circular wall.

The isothermal lines become more and more distorted with enhances of the Rayleigh number. At the higher Rayleigh number ($Ra=10^{6}$), the streamlines form a particular pattern like a mushroom. This particular pattern of the streamlines indicates that the heat energy flows into the nanofluid within the enclosure from the heated bottom wall. For the increases of Rayleigh number (*Ra*), the isothermal lines are more distorted at the middle of the heated wall, indicating convection is beginning to take over and become a dominant mode of heat transport within the enclosure.

### 5.4 Effect of uniform/non-uniform thermal boundary condition (TBC)

**Figure 11:**
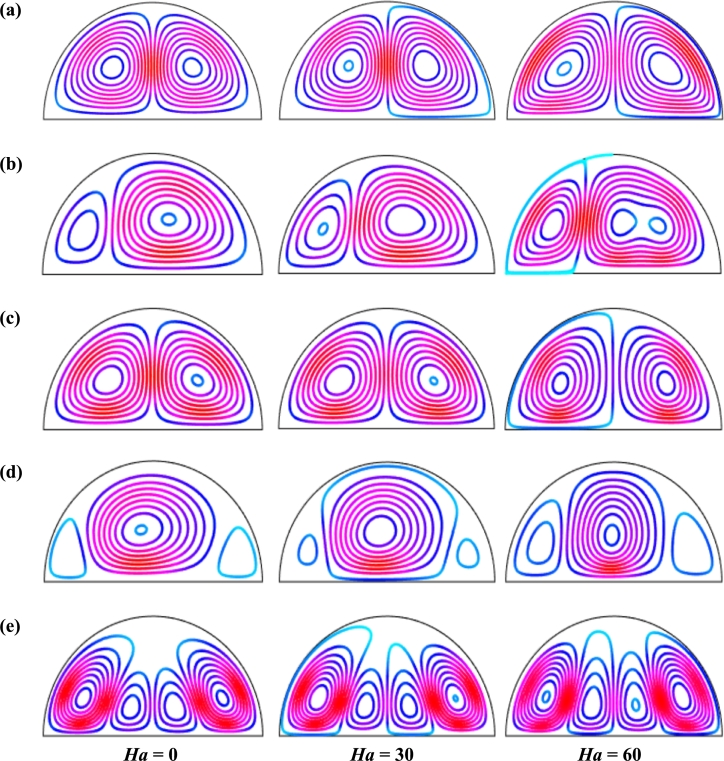
Streamlines of *Ha* for TBC (a) *θ* = 1 (case I), (b) *θ* = 1 − *X* (case II), (c) *θ* = *X* (1 − *X*) (case III), (d) θ=Asin⁡(2πX) (case IV) and (e) *θ* = *A*sin^2^ (2*πX*) (case V) with *Ra* = 10^5^, *ϕ* = 0.04, *d* = 10 nm, *λ* = 0.5, *n* = 3 and *τ* = 1 (steady case).

**Figure 12:**
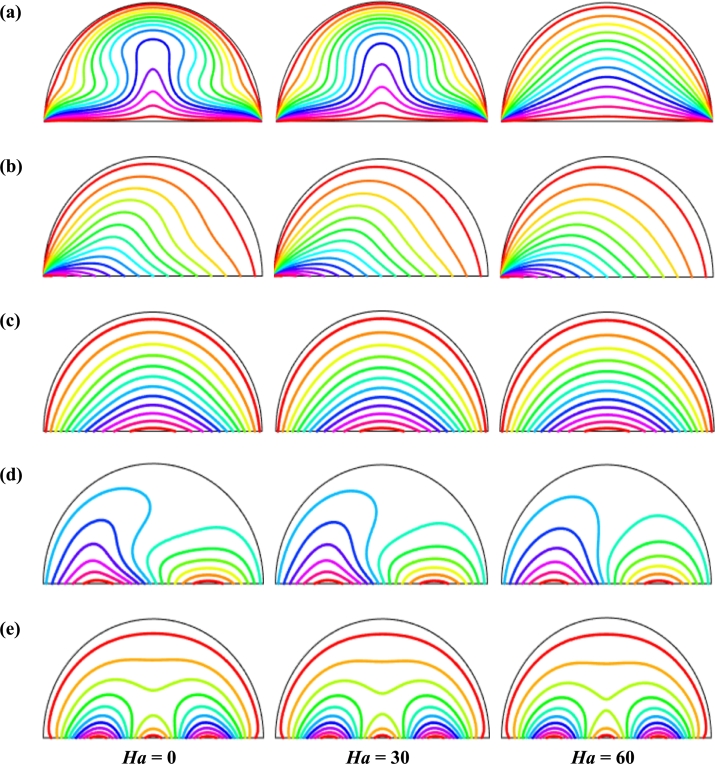
Isotherms of Hartmann number (*Ha*) for different thermal boundary conditions (a) *θ* = 1 (case I), (b) *θ* = 1 − *X* (case II), (c) *θ* = *X* (1 − *X*) (case III), (d) θ=Asin⁡(2πX) (case IV) and (e) *θ* = *A*sin^2^ (2*πX*) (case V) for Cu-H_2_O nanofluid with *Ra* = 10^5^, *ϕ* = 0.04, *d* = 10 nm, *λ* = 0.5, *n* = 3, and *τ* = 1 (steady case).

**Figure 13:**
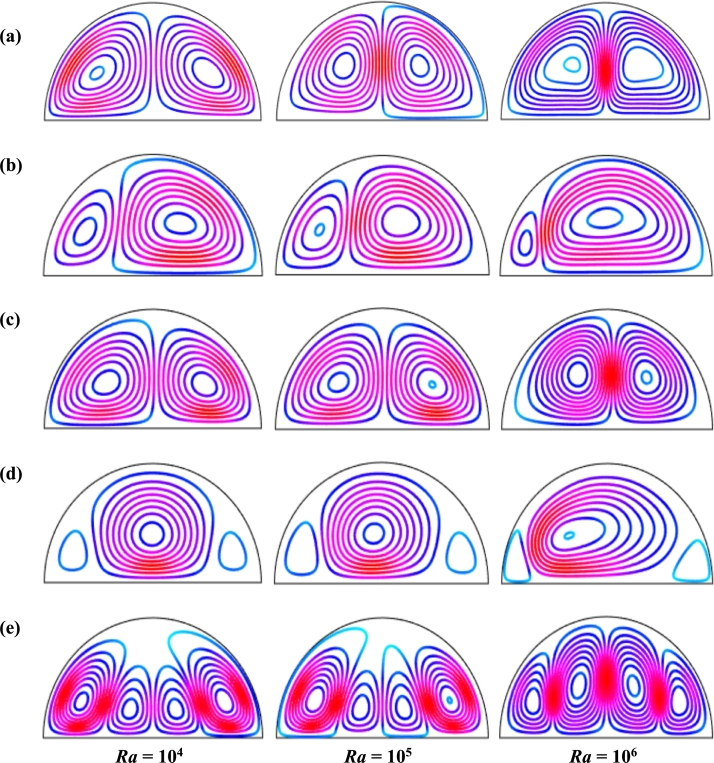
Streamlines of Rayleigh number (*Ra*) for different thermal boundary conditions (a) *θ* = 1 (case I), (b) *θ* = 1 − *X* (case II), (c) *θ* = *X* (1 − *X*) (case III), (d) θ=Asin⁡(2πX) (case IV) and (e) *θ* = *A*sin^2^ (2*πX*) (case V) for Cu-H_2_O nanofluid when *Ha* = 20, *ϕ* = 0.04, *d* = 10 nm, *λ* = 0.5, *n* = 3, and *τ* = 1 (steady case).

**Figure 14:**
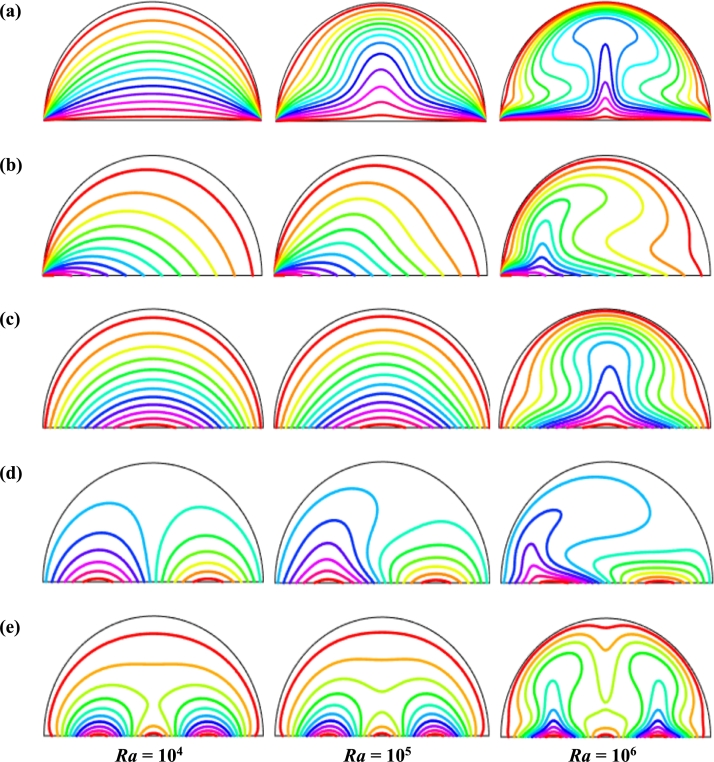
Isotherms of Rayleigh number (*Ra*) for different thermal boundary conditions (a) *θ* = 1 (case I), (b) *θ* = 1 − *X* (case II), (c) *θ* = *X* (1 − *X*) (case III), (d) θ=Asin⁡(2πX) (case IV) and (e) *θ* = *A*sin^2^ (2*πX*) (case V) for Cu-H_2_O nanofluid when *Ha* = 20, *ϕ* = 0.04, *d* = 10 nm, *λ* = 0.5, *n* = 3, and *τ* = 1 (steady case).

**Figure 15:**
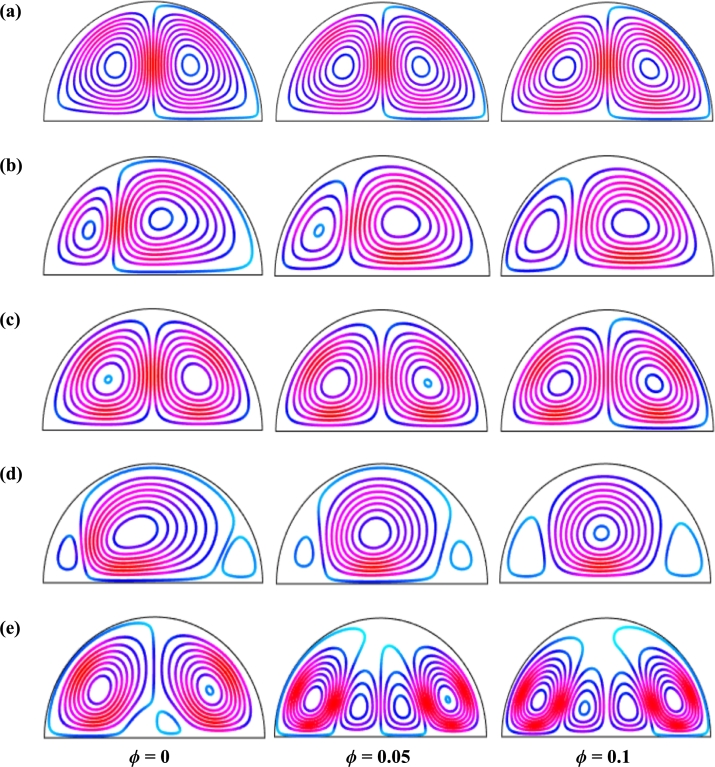
Streamlines of volume fraction of nanoparticles (*ϕ*) for different thermal boundary conditions (a) *θ* = 1 (case I), (b) *θ* = 1 − *X* (case II), (c) *θ* = *X* (1 − *X*) (case III), (d) θ=Asin⁡(2πX) (case IV) and (e) *θ* = *A*sin^2^ (2*πX*) (case V) for Cu-H_2_O nanofluid when *Ha* = 20, *Ra* = 10^5^, *d* = 10 nm, *λ* = 0.5, *n* = 3, and *τ* = 1.

**Figure 16:**
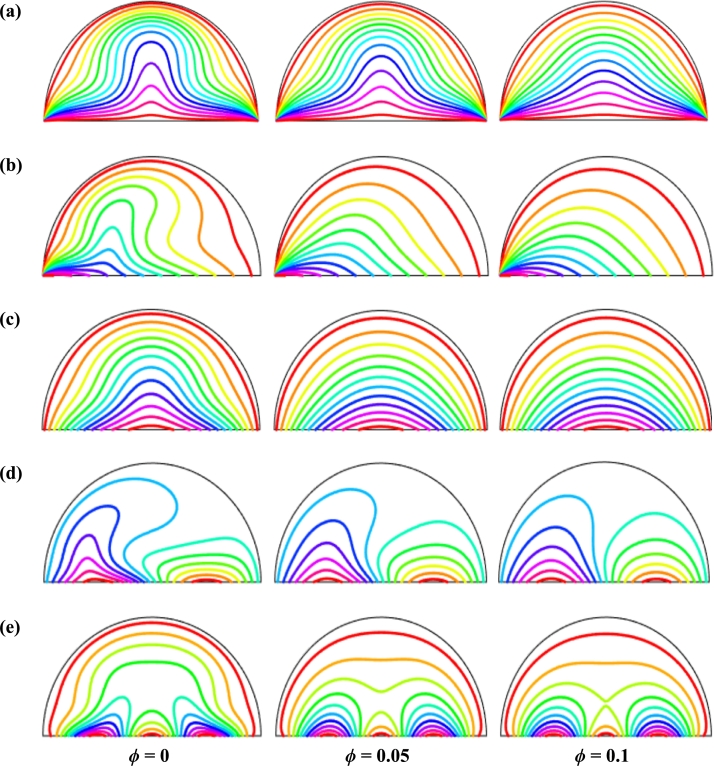
Isotherms of nanoparticles volume fraction (*ϕ*) for different thermal boundary conditions (a) *θ* = 1 (case I), (b) *θ* = 1 − *X* (case II), (c) *θ* = *X* (1 − *X*) (case III), (d) θ=Asin⁡(2πX) (case IV) and (e) *θ* = *A*sin^2^ (2*πX*) (case V) for Cu-H_2_O nanofluid when *Ha* = 20, *Ra* = 10^5^, *d* = 10 nm, *λ* = 0.5, *n* = 3, and *τ* = 1.

Figure 11, Figure 12, Figure 13, Figure 14, Figure 15, Figure 16 show the numerical outcomes of natural convective temperature transport inside a half-moon shaped domain with uniform/non-uniform thermal boundary conditions (case I: θ=1 (uniformly heated), case II: θ=1−X (linearly heated), case III: θ=X(1−X) (parabolically heated), case IV: θ=A sin(2*πX*) (sinusoidally heated), and case V: $\theta =A\sin^{2}(2\pi X)$ (square of sinusoidally heated)). The characteristics of controlling parameters such as Rayleigh number (*Ra*), Hartmann number (*Ha*), and nanoparticles volume fraction (*ϕ*) are investigated on the physical phenomenon of the flow field. For numerical simulation, Cu-H_2_O nanofluid is considered as default nanofluid.

Figure 11, Figure 12 display the effects of streamlines and isothermal lines respectively for different Hartmann numbers (*Ha*) when the bottom wall is heated uniformly and non-uniformly. The fluid near the bottom diameter is hotter compared to the circular wall. So, the fluid near the bottom wall has a lower density compared to the fluid near-circular wall. Consequently, the fluid near the middle of the bottom wall moves upward while relatively heavy fluid near the circular wall moves downward along the circular wall. As a result, the fluid loss energy moves downward and eventually forces the separation of the thermal boundary layer along the circular wall. In Fig. 11, for uniformly thermal boundary conditions (case I), two counterclockwise central circulation cells are observed within the enclosure. The eye of rotations is situated near the center of each half of the cross-section of the enclosure. But for non-uniform thermal boundary conditions (case V), four counter-clockwise rotating vortices are observed. Also, two symmetrical rotating circulation cells are observed for the absence of magnetic force (Ha=0). But, for applying magnetic force introduced by Hartmann number (Ha=60), it is seen that the movement of the fluid become slower within the enclosure compared to the case of Hartmann number (Ha=0) because the magnetic field has a trend to make slowdown the motion of the fluid. The symmetry also changes for the increase of Hartmann number for case III and case V because the magnetic field suppresses the circulation of the flow within the enclosure. A large rotating cell is seen for a higher magnetic field in case II and case IV.

The isothermal contours of Hartmann number (*Ha*) for various thermal boundary conditions are presented in Fig. 12. These figures show that the isothermal lines are clustered with the heated bottom wall, indicating the existence of a temperature gradient along the vertical direction of this region. It is also observed that the temperature gradient is weak at the center of the cavity. At higher Hartmann number (Ha=60), the isothermal lines are almost parallel to each other in case I, case IV, and case V. The isothermal lines are densely distributed at the left corner inside the cavity in case II. In contrast, isotherms are densely distributed at both the bottom corner of the enclosure for case I and case III. In case I, the isothermal lines are formed a mushroom shape at the middle of the cavity for low Hartmann Number. For the consideration of Lorentz forces, i.e., an increase of Hartmann number, the isothermal lines start to move away from the hot bottom wall, indicating temperature gradient decreases within the enclosure.

Figure 13, Figure 14 represents the impact of streamlines and isothermal lines, respectively for different Rayleigh number (*Ra*) with various thermal boundary conditions. For uniform thermal boundary conditions (case I) and non-uniform thermal boundary conditions (case III), two counter symmetrical circulation cells are observed inside the cavity, and the eye of the rotation is located near the center of each cross-section of the cavity. But the symmetry is distorted with the increase of Rayleigh number. At a higher Rayleigh number, ($Ra=10^{6}$), the convection is more pronounced than conduction. A large central circulation cell with two small tubes at the corner of the bottom wall is observed at the center of the cavity for the sinusoidal thermal boundary condition (case IV). The density of the streamlines enhances within the enclosure with the increase of Rayleigh number (*Ra*) due to the convection mode of heat transfer dominates in those regions. For non-uniform thermal boundary conditions (case V), there are four symmetric circulating cells are also observed. Two larger cells at the middle of the enclosure are observed for a higher Rayleigh number ($Ra=10^{6}$) in case V. Besides these two primary cells, two secondary cells are formed at the corner of the heated bottom wall.

To detect the effectiveness of temperature transfer, isothermal lines are useful. The isothermal lines also help us to detect the mode of temperature transport, whether it is conduction or convection. Fig. 14 shows that isothermal lines are more compressed near the bottom wall. These compressed isothermal lines tell us that the principal mode of heat transport is conduction at those regions. At the middle of the cavity, the density of the isothermal lines is less, which represents relatively weaker convective heat transfer. Fig. 14(a) represents uniform heating at the bottom wall causes a finite discontinuity in Dirichlet type of boundary conditions for the distribution of the temperature at both edges of the bottom wall. For non-uniform heating (case IV and case V), the singularity is removed at the edges of the bottom wall. The isothermal lines are quite dispersed throughout the cavity for a relatively higher Rayleigh number. For sinusoidal thermal boundary conditions, the dispersion of isothermal lines increases within the half-moon-shaped domain. Fig. 14(a) shows that isothermal lines are distributed uniformly, representing conduction as the principal mode of heat transport.

For the higher *Ra*, the isothermal lines are more distorted due to the more substantial effects of convection. At $Ra=10^{6}$, case I and case III, the isothermal lines form like a mushroom shape at the middle of the cavity, indicating convection is dominant at that region. Thus, a higher value of *Ra* improves the convection heat transfer characteristics.

Figure 15, Figure 16 depict the impact of streamlines and isotherms, respectively for different nanoparticles volume fraction (*ϕ*) with various thermal boundary conditions for Cu-H_2_O nanofluid when Ha=20, $Ra=10^{5}$, d=10 nm, λ=0.5, n=3, and τ=1. These figures show that both streamline contours and isothermal lines are affected significantly with the increases of nanoparticles volume fraction in all thermal boundary conditions (case I, II, III, IV & V). The pattern of the streamline contours is almost similar for the thermal system of the case I & III. A central large rotating vortex with two small eddies is observed for sinusoidal thermal boundary conditions (case IV). The streamline contours are expanded, and the little eddies become stronger within the cavity with the increases of the volume of nanoparticles. This figure shows that three symmetrical parallel distributions of the family of curves of isothermal lines from bottom diameter for the entire range of nanoparticles volume in case V. The thickness of the isothermal lines increases with the increment of nanoparticles volume fraction.

Table 2 represents the average Nusselt number on the heated bottom wall with various thermal boundary conditions for Cu-H_2_O nanofluid when Pr=6.84 and ϕ=0.04. To determine the rate of heat transfer along the bottom heated wall for engineering applications, the average Nusselt number is calculated varying diameter of nanoparticles (*d*), Rayleigh number (*Ra*), and Hartmann number (*Ha*) regarding uniform thermal boundary condition (case I), linear thermal boundary condition (case II), non-uniform thermal boundary condition (case III, IV, & V). This Table shows that the average Nusselt number decreases with the increase of Hartmann number (*Ha*). Therefore, the rate of heat transfer is reduced by the stronger magnetic field. This Table also shows that the heat transport is intensified for copper-water nanofluid by decreasing the size of nanoparticles and higher Rayleigh number (*Ra*). This table shows that heat transport rate decreases 1.55% with $Ra=10^{4}$, whereas it decreases 21.14% with $Ra=10^{6}$ when *Ha* varies 0 to 80 with d=1 nm and uniform thermal system (case I). In addition, it is interesting to observe that height heat transmission is achieved when the bottom wall is heated uniformly. The temperature transport rate increases 19.53% for the case I, 18.73% for case II, 0.95% for case III, 10.56% for case IV, and 4.95% for case V when *Ra* varies 10^4^ to 10^6^ when Ha=80, and d=1 nm.

**Table 2:**
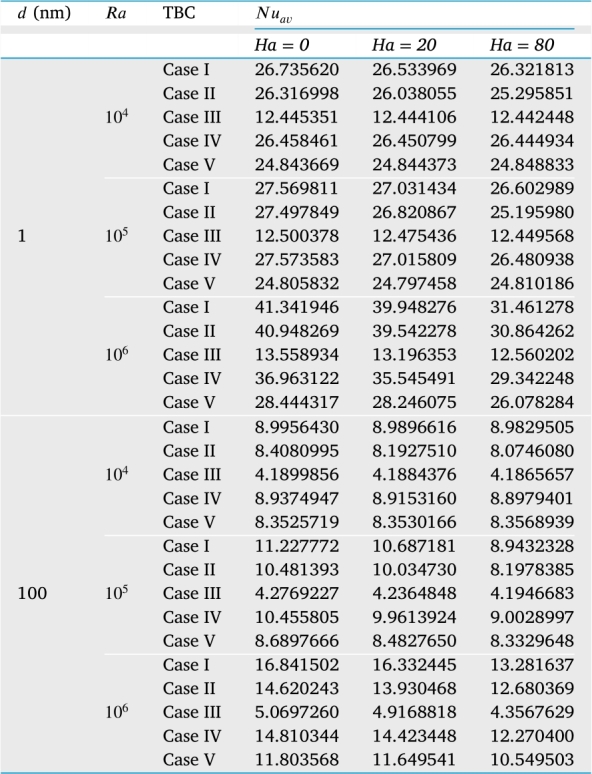
Variation of *Nu*_*av*_ for Cu-H_2_O nanofluid along the bottom heated wall with various thermal boundary conditions (TBC) when *Pr* = 6.84, *n* = 3, *ϕ* = 0.04, and *τ* = 1.

### 5.5 Effect of nanoparticle volume fraction

**Figure 17:**
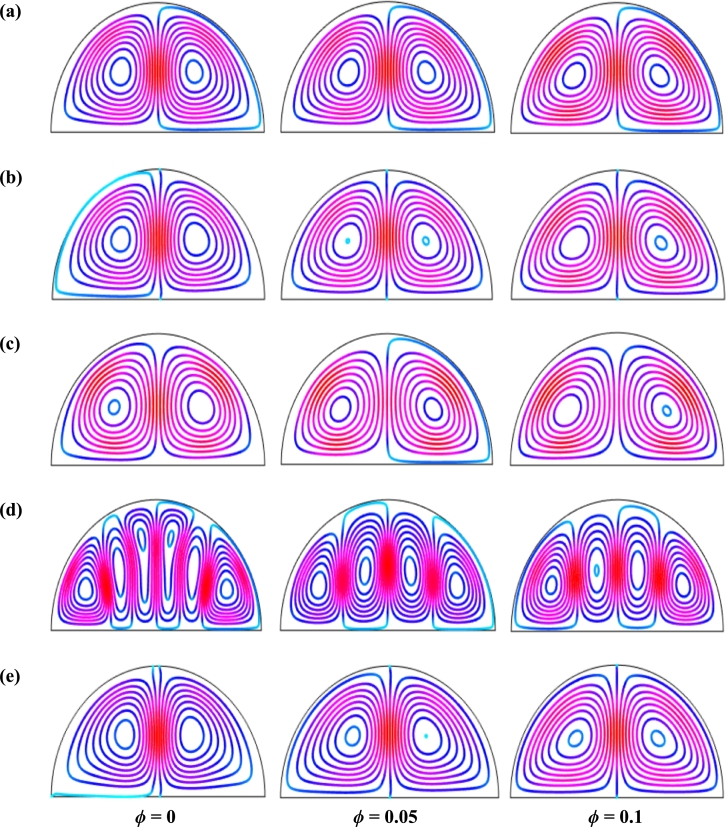
Effect of nanoparticles volume fraction (*ϕ*) on streamlines for (a) uniform magnetic field (umf), (b) *λ* = 0.1, (c) *λ* = 0.25, (d) *λ* = 0.5, and (e) *λ* = 1, for Cu-H_2_O nanofluid with uniform thermal boundary condition when *Ha* = 20, *Ra* = 10^5^, *d* = 10 nm, *n* = 3, and *τ* = 0.1 (steady case).

Fig. 17 represents the impact of streamlines for different nanoparticles volume fraction for Cu-H_2_O nanofluid for uniform thermal boundary condition (case I) when Pr=6.84, Ha=20, d=10 nm, $Ra=10^{5}$, and n=3 for unsteady case τ=0.1. At the non-dimensional time, τ=0.1, it is observed that there are two opposite circulation cells within the cavity for both uniform magnetic field and non-uniform magnetic field. Thermal boundary heating condition is the causes for the pattern of this streamlines. The nanofluids near the bottom wall become heated by the heated bottom wall and move upwards while the bottom relatively cold nanofluid near-circular wall approaches the bottom wall, which helps to create a symmetrical flow pattern. This pattern remains the same for the categorical of nanoparticles volume fraction (*ϕ*). An interesting observation is that the addition of nanoparticles into the base fluid decreases the value of stream function for a particular period of the magnetic field. The cause behind this phenomenon is that the addition of nanoparticles enhances the total mass of the fluid within the cavity which increases the inertia force of the fluid. The flow of the fluid becomes to slow down slightly for this higher inertia. The pattern of streamlines is almost similar for both uniform magnetic fields (case (a)) and low periods (λ=0.1) of the magnetic field (case (b)). The flow pattern changes with the increases of the magnetic field period, while an interesting pattern of the streamlines is seen for the period of the magnetic field, λ=0.5.

**Figure 18:**
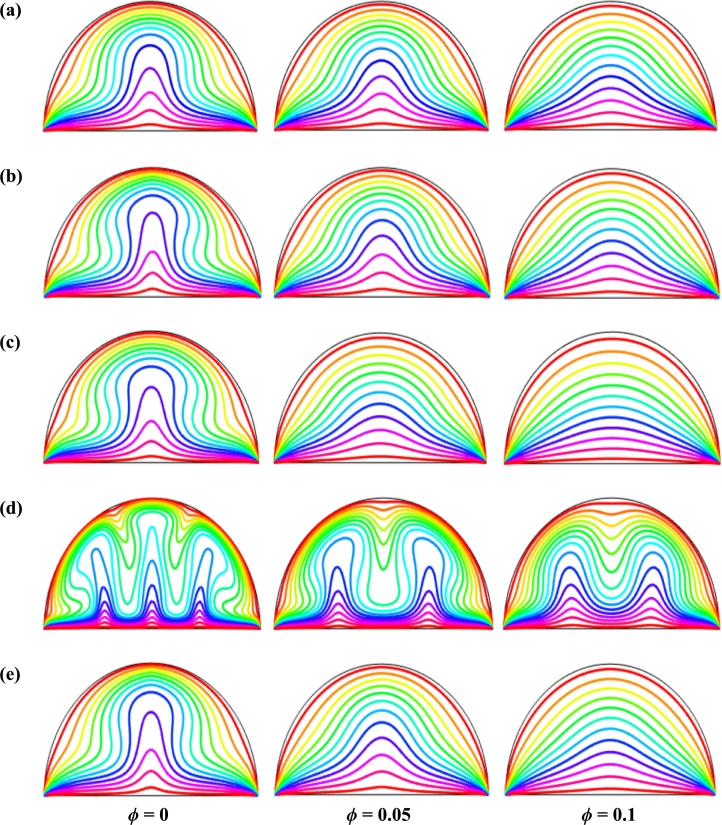
Effect of *ϕ* on isotherms for (a) uniform magnetic field (umf), (b) *λ* = 0.1, (c) *λ* = 0.25, (d) *λ* = 0.5, and (e) *λ* = 1, for Cu-H_2_O nanofluid with uniform thermal boundary condition when *Ha* = 20, *Ra* = 10^5^, *d* = 10 nm, *n* = 3, and *τ* = 0.1 (steady case).

The pattern of isothermal lines for different value of volume fraction of the nanoparticles is presented in Fig. 18 at the unsteady case τ=0.1 for Cu-H_2_O nanofluid for uniform thermal boundary condition (case I) when Pr=6.84, Ha=20, d=10 nm, $Ra=10^{5}$, and n=3. This figure shows that the isothermal lines are more compressed near the corner of the bottom diameter. The closely packing of the streamlines indicate that conduction is the major type of heat transport. The density of the streamlines is lower in the middle of the bottom diameter, which indicates a higher heat transport region. The isothermal lines pattern changes with the addition of the nanoparticles. In addition, the addition of nanoparticles into the base fluid has a stimulating effect on heat diffusion. The pattern of the isothermal lines changes with the changes of the period of the magnetic field.

The study also investigated the effects of governing physical parameters like as diameter of the nanoparticles (*d*), nanoparticles volume fraction (*ϕ*), Hartmann number (*Ha*), the different shape factor of nanoparticles (*n*), Brownian motion of the nanoparticles, and Rayleigh number (*Ra*) of different nanofluids on the rate of heat transfer at the heated bottom wall of the cavity. This goal is satisfied by Table 3. The results of the present problem are discussed for copper-water nanofluid. Various types of nanoparticles and base fluid are also considered in the present study to observe how the outcomes depend on them. Table 3 illustrates the average Nusselt number at the heated wall of the cavity for different types of nanofluids and various value of nanoparticles volume fractions for uniform thermal boundary condition (case I) for four different types of base fluids such as water (H_2_O), kerosene, ethylene glycol (EG) and engine oil (EO) with eight different types of nanoparticles such as Cu, Co, Fe_3_O_4_, Al_2_O_3_, TiO_2_, Ag, Zn, and CuO when $Ra=10^{5}$, Ha=20, d=10 nm, and τ=1. It is seen that the temperature changes are significant despite nanoparticles providing 1% into the base fluid. The heat transfer rate increases 5.4% for engine oil-based nanofluid and 36.89% for ethylene glycol-based nanofluid with the increase of 1% nanoparticles volume.

**Table 3:** Average Nusselt number at the bottom heated wall with the case I for different types of nanofluids and nanoparticles volume fractions when *Ra* = 10^5^, *Ha* = 20, *d* = 10 nm, *n* = 3, and *τ* = 1.

Nanofluids	*ϕ*						
	I	II	$\left(\frac{\mathrm{II}-I}{I}\right)\times 100$	III	$\left(\frac{\mathrm{III}-I}{I}\right)\times 100$	IV	$\left(\frac{\mathrm{IV}-I}{I}\right)\times 100$
	0	0.01		0.05		0.1	
Cu-water	8.40238	10.26319	22.15	16.069668	91.25	23.0502	174.33
Co-water	8.40238	10.40006	23.78	17.094337	103.45	24.4121	190.54
Fe_3_O_4_-water	8.40238	10.34482	23.12	17.119632	103.75	34.3054	308.28
Ag-water	8.40238	9.795710	16.58	14.577452	73.49	19.5149	132.25
Zn-water	8.40238	9.93067	18.19	15.129334	80.06	20.612	145.31
CuO-water	8.40238	10.20305	21.43	16.232929	93.19	22.817	171.55
Al_2_O_3_-water	8.40238	10.02129	19.27	15.403518	83.32	21.327	153.82
TiO_2_-water	8.40238	9.939298	18.29	15.051253	79.13	20.5714	144.84
Cu-kerosene	8.45536	13.77058	62.86	32.387029	283.04	54.6207	545.99
Co-kerosene	8.45536	14.19283	67.86	34.469768	307.67	58.6155	593.23
Fe_3_O_4_-kerosene	8.45536	13.80784	63.30	32.608736	285.66	55.0388	550.93
Ag-kerosene	8.45536	12.35142	46.08	25.610078	202.89	41.3650	389.22
Zn-kerosene	8.45536	12.77932	51.14	27.656223	227.07	45.5296	438.47
CuO-kerosene	8.45536	13.6266	61.16	31.766445	275.69	53.5183	532.95
Al_2_O_3_-kerosene	8.45536	13.13515	55.35	29.443429	248.22	49.1617	481.43
TiO_2_-kerosene	8.45536	12.95848	53.26	28.575965	237.96	47.4631	461.34
Cu-EO	8.40664	8.860633	5.400	10.548415	25.48	12.4041	47.55
Co-EO	8.40664	8.883704	5.680	10.654252	26.74	12.5938	49.81
Fe_3_O_4_-EO	8.40664	8.869217	5.500	10.628576	26.43	12.6495	50.46
Ag-EO	8.40664	8.790590	4.570	10.221352	21.59	11.8116	40.50
Zn-EO	8.40664	8.796323	4.640	10.255262	21.99	11.8793	41.31
CuO-EO	8.40664	8.838539	5.140	10.447394	24.28	12.2218	45.38
Al_2_O_3_-EO	8.40664	8.795464	4.630	10.238918	21.79	11.8265	40.68
TiO_2_-EO	8.40664	8.779831	4.440	10.164708	20.93	11.6837	38.98
Cu-EG	8.53059	11.67756	36.89	21.899593	156.72	33.6379	294.32
Co-EG	8.53059	11.92407	39.78	22.946893	168.99	35.7585	319.18
Fe_3_O_4_-EG	8.53059	11.71798	37.36	22.146809	159.62	33.9620	298.12
Ag-EG	8.53059	10.84633	27.15	18.446205	116.24	26.6427	212.32
Zn-EG	8.53059	11.09985	30.12	19.469056	128.23	28.7921	237.52
CuO-EG	8.53059	11.59676	35.94	21.531137	152.40	33.0192	287.07
Al_2_O_3_-EG	8.53059	11.3093	32.57	20.278709	137.72	30.6308	259.07
TiO_2_-EG	8.53059	11.19740	31.26	19.807656	132.20	29.6401	247.46

### 5.6 Effect of nanoparticles diameter

**Figure 19:**
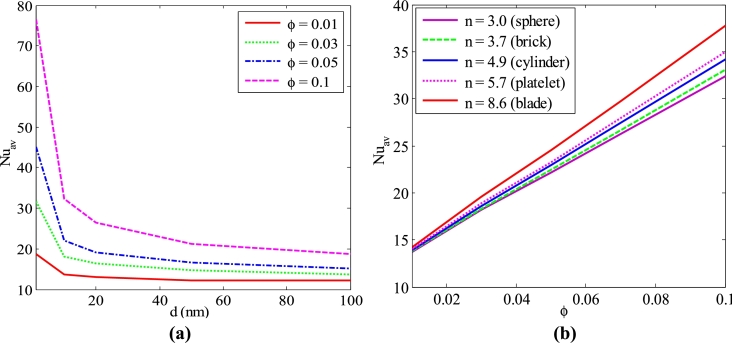
*Nu*_*av*_ for different (a) nanoparticles volume fraction (*ϕ*) and (b) shape (n) of nanoparticles diameter (*d*) for Cu-H_2_O nanofluid having uniform thermal boundary condition (case I) with *Ra* = 10^5^, *Ha* = 20, *n* = 3, and *τ* = 1.

Fig. 19 (a-b) shows the mean Nusselt number for various nanoparticles volume fractions (*ϕ*) and shape of particles such as spherical, blade, platelet, cylinder, and brick shape against different diameters of nanoparticles (*d*) and solid concentration, respectively for Cu-H_2_O nanofluid with the uniform thermal system (case I) when Pr=6.84, $Ra=10^{5}$, λ=0.5, Ha=20, and τ=1 (steady-state). The graph shows that the average Nusselt number is decreased for the increase in the diameter of the nanoparticles. The significant changes of the average Nusselt number happen for about 1-50 nm particles size. After that, it remains almost similar for the 51-100 nm size of particles in the solution. In addition, the average Nusselt number is significantly higher for the 1-10 nm size of nanoparticles. It is noticed that the mean Nusselt number is more pronounced and intensified for a higher volume fraction of nanoparticles. In addition, a decreasing trend of heat transfer rate is seen for the increases of the diameter of nanoparticles. Furthermore, the nanoparticles move the cold upper wall because of the temperature gradient between hot and cold walls. These outcomes indicate that as the temperature gradient increases, the diffusion of the nanoparticles also increases, which increases the heat transfer rate. The nanoparticles of spherical shape have been considered for the numerical outcomes of the present problem. Different sizes of nanoparticles are also used in the current investigation for analyzing the shape effects on heat transport. Fig. 19(b) depicts that the rate of heat transfer is significantly higher for the blade shape of nanoparticles than the spherical shape of nanoparticles. This is because of the less sphericity of the blade shape of nanoparticles. The physical meaning of the higher total surface area of the blade-shaped solid-liquid crossing point is associated with the whole external area of the sphere-shaped nanoparticle-liquid edge for the same amount of volume fraction of nanoparticles. In addition, the average Nusselt number is more apparent for the higher value of nanoparticle volume fraction.

### 5.7 Effect of nanoparticles shape factor

The average Nusselt number is higher for the blade shape of nanoparticles compared to all other shapes of nanoparticles such as spherical, brick, cylinder, and platelet. It is also clear from this figure that a classification of the performance of heat transport from higher performance to lesser performance concerning nanoparticles size is the blade, platelet, cylinder, brick, and spherical shape, respectively. Table 4 shows the impact of the size of the nanoparticles for different types of nanofluid for uniform thermal boundary conditions (case I). For all types of nanofluids, the blade shape of nanoparticles shows a higher heat transfer rate than other shapes of nanoparticles. The rate of heat transport increases 10.43% blade shape of nanoparticles instead of the spherical shape of nanoparticles for Cu-H_2_O nanofluid with uniform thermal boundary condition.

**Table 4:**
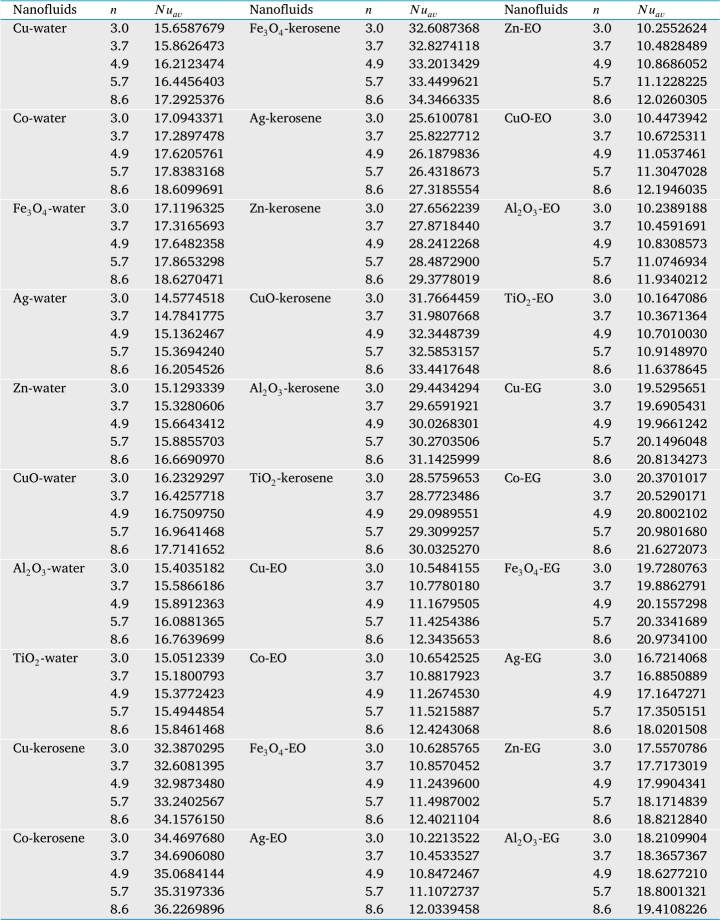
*Nu*_*av*_ at bottom heated wall (case I) for different types of nanofluids and shape of nanoparticles with *Ra* = 10^5^, *Ha* = 20, *d* = 10 nm, and *τ* = 1.

### 5.8 Brownian motion in thermal conductivity

The impact of Brownian motion of nanoparticles has been considered in the thermal conductivity equation for calculating all results, as shown in equations (18)-(19). The heat transport rate regarding the average Nusselt number along the heated diameter has been calculated for examining the influence of Brownian motion on the rate of heat transfer. The outcome indicates Brownian motion plays an influential role in the augmentation of temperature transport rate. Due to the movement of the nanoparticles into the base fluid, the Brownian motion of the nanoparticles contributes to the transfer more heat in the nanofluids and micro-convection of the fluid around individual nanoparticles. The result shows that at low Rayleigh number, the impact of nanoparticles volume fraction on average Nusselt number is more effective for both cases without and with Brownian motion of the nanoparticles. In addition, for higher nanoparticles volume fraction (ϕ=0.01), the average heat transport is increased by 28.88% considering Brownian motion of the nanoparticles at low Rayleigh number ($Ra=10^{4}$) and by 3.01% when nanoparticles Brownian motion is neglected.

Table 5 depicts the average Nusselt number on the heated bottom wall of the enclosure for different types of nanofluids and different diameters of nanoparticles for uniform TBC of case I for four different types of base fluids such as water (H_2_O), kerosene, ethylene glycol (EG) and engine oil (EO) with eight different types of nanoparticles such as Cu, Co, Fe_3_O_4_, Al_2_O_3_, TiO_2_, Ag, Zn, and CuO when $Ra=10^{5}$, Ha=20, d=10 nm and τ=1. This Table shows that the average rate of heat transfer decreases with the increase of the diameter of nanoparticles. Kerosene-based nanoparticles show significant augmentation in heat transfer rate. Engine oil-based nanofluids show a lower rate of heat transfer. This is because engine oil has higher dynamical viscosity, which suppresses the nanoparticle's Brownian motion. Therefore, kerosene-based nanofluids show a higher heat transfer rate compared to water-based nanofluids. In addition, by decreasing nanoparticles diameter, the specific area increases, which helps to enhance nanofluid thermal conductivity and consequently increases the average Nusselt number. This Table shows that the rate of heat transfer rate increases 97.17% in kerosene-cobalt nanofluid whereas it increases 8.61% for engine oil-based cobalt nanoparticles when nanoparticles diameter decreases from 100 nm to 10 nm. In addition, for addition nanoparticles volume (ϕ=0.01), the average Nusselt number at the heated wall is increased by 28.88% with 1% nanoparticles volume at low Rayleigh number ($Ra=10^{4}$) when Brownian motion of the nanoparticles is considered into account and by 3.01% when Brownian motion of the nanoparticles is neglected.

**Table 5:**
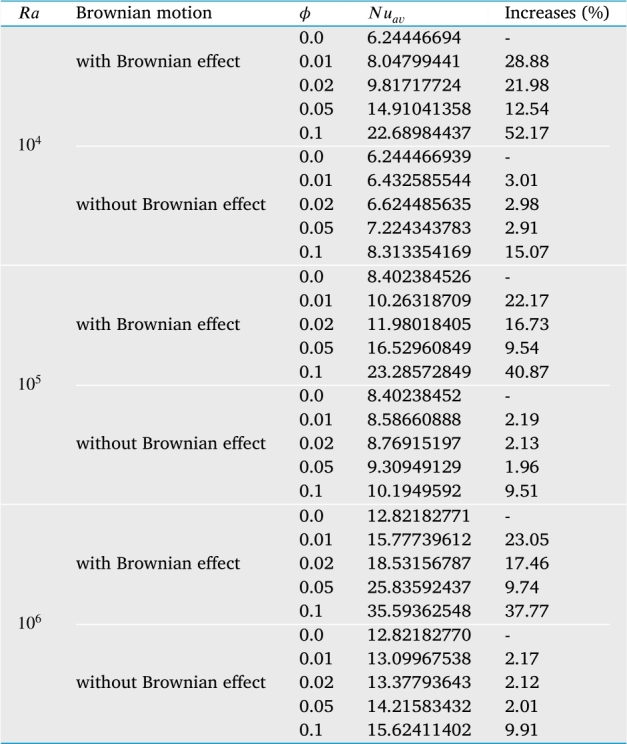
*Nu*_*av*_ at the heated wall with the case I for different values of nanoparticles volume fraction and Rayleigh number for “without Brownian motion” effects and “with Brownian motion” effects for Cu-H_2_O nanofluid when *Ha* = 20, *Pr* = 6.84, *n* = 3, and *τ* = 1.

### 5.9 Comparison

The present numerical result has been compared concerning the average Nusselt number with previously published work which is included in Table 6. The numerical outcomes of mean Nusselt number for the effect of the nanoparticles volume fraction and Rayleigh number considering uniformly heated bottom wall and uniform magnetic field intensity are compared with Ghasemi et al. [10] for the steady-state case. The two-dimensional physical problem about the natural convective flow of Al_2_O_3_-water nanofluid within a square cavity with the existence of a horizontally magnetic effect was investigated by Ghasemi et al. [10]. The range of the numerical values of the solid concentration and Rayleigh number are 0≤ϕ≤0.04 and $10^{3}\leq Ra\leq 10^{7}$, respectively, and Ha=30, Pr=6.84 are kept fixed. The uniform thermal boundary condition (case I) and the uniform magnetic field is considered for this comparison. The comparison shows very good conformity of the present result with [10].

**Table 6:** Comparison of *Nu*_*av*_ for different *Ra* and *ϕ* with Ghasemi et al. [10].

Ra	ϕ=0		ϕ=0.02		ϕ=0.04	
	Ghasemi et al. [10]	Present Study	Ghasemi et al. [10]	Present Study	Ghasemi et al. [10]	Present Study
10^3^	1.002	1.002	1.060	1.060	1.121	1.121
10^4^	1.183	1.182	1.212	1.208	1.249	1.242
10^5^	3.150	3.138	3.138	3.097	3.124	3.057
10^6^	7.907	7.820	7.979	7.796	8.042	7.773
10^7^	16.929	16.317	17.197	16.992	17.449	16.865

## 6 Conclusions

The leading purpose of this investigation is to perform the influence of non-uniform vertically periodic magnetic field on time-dependent two-dimensional, laminar, incompressible flow and heat transport enhancement considering nanoparticles Brownian motion of nanofluids inside a semi-circular cavity. The function of the magnetic effects has been considered as the sine function of *x*. However, both uniform and non-uniform magnetic fields have also been examined in this work. The upper circular wall has been cooled at low temperature, while the horizontal bottom diameter is heated at high temperature. Different types of nanofluids and thermal boundary conditions are also examined to investigate the natural convection heat transport mechanism. The outcomes for different physical model parameters such as Hartmann number (*Ha*), Rayleigh number (*Ra*), the diameter of nanoparticles (*d*), the volume fraction (*ϕ*), size (*n*), period of the uniform/non-uniform magnetic field (*λ*), and Brownian motion of the nanoparticles have been displayed using streamlines, isotherms and the average rate of heat transport. The numerically simulated results are compared with previously published work and found an excellent agreement. A comprehensive discussion of these physical parameters has been done from the physical point of view. The important findings are listed as follows:

• The heat transfer mechanism of the solution for Cu-H_2_O nanofluid reaches a steady-state from the unsteady situation within a very short time about τ=0.65 for uniform thermal boundary condition (case I).
• Stronger uniform (λ=0) magnetic field intensity decreases thermal transport compared to the lower intensity.
• The non-uniform magnetic field shows a higher heat transfer rate along the heated wall than the uniform case and the highest heat transport rate is noticed for the period λ=0.75.
• The higher Rayleigh number confirms better temperature transfer in natural convection. Heat transfer rate increases 56.30% when *Ra* varies from 10^5^ to 10^6^.
• The thermal boundary conditions have an effective role in fluid flow and heat transport. The uniform thermal condition (case I) provides the highest heat transport rate than other thermal conditions (case II, III, IV, V) for copper-water nanofluid.
• The concentration of nanoparticles improves $Nu_{av}$ by approximately 22.14% for Cu-H_2_O nanofluid even the addition is 1% than water.
• The diameter of nanoparticles has a significant effect on making the solution of the nanofluid stable. The small size of nanoparticles assists in enhancing the thermal conductivity of nanofluids.
• The blade-shaped nanoparticle shows a higher heat transfer rate of about 5.10% than spherical-shaped for Co-kerosene nanofluid.
• The rate of thermal transport is observed as 67.86% for Co-kerosene, whereas 23.78% for Co-water, 5.67% for Co-engine oil, and 39.78% for Co-ethylene glycol with an increase of 1% nanoparticles volume fraction with base fluids.
• The heat transfer rate increases about 22.17% with the Brownian activity, whereas it enhances about 2.19% without the Brownian motion using 4% concentrated Cu-H_2_O nanofluid than water at $Ra=10^{5}$.

## Declarations

### Author contribution statement

Tarikul Islam: Conceived and designed the experiments; Performed the experiments; Analyzed and interpreted the data; Contributed reagents, materials, analysis tools or data; Wrote the paper.

N. Parveen: Conceived and designed the experiments.

R. Nasrin: Conceived and designed the experiments; Analyzed and interpreted the data; Contributed reagents, materials, analysis tools or data; Wrote the paper.

### Funding statement

This research did not receive any specific grant from funding agencies in the public, commercial, or not-for-profit sectors.

### Data availability statement

Data included in article/supp.material/referenced in article.

### Declaration of interests statement

The authors declare no conflict of interest.

### Additional information

No additional information is available for this paper.
